# Virulence of the Pathogen Porphyromonas gingivalis Is Controlled by the CRISPR-Cas Protein Cas3

**DOI:** 10.1128/mSystems.00852-20

**Published:** 2020-09-29

**Authors:** Jose Solbiati, Ana Duran-Pinedo, Fernanda Godoy Rocha, Frank C. Gibson, Jorge Frias-Lopez

**Affiliations:** a Department of Oral Biology, College of Dentistry, University of Florida, Gainesville, Florida, USA; University of Georgia

**Keywords:** CRISPR-Cas, virulence, pathogenesis, periodontal disease, *Porphyromonas gingivalis*

## Abstract

The CRISPR (clustered regularly interspaced short palindromic repeat)-Cas system is a unique genomic entity that provides prokaryotic cells with adaptive and heritable immunity. Initial studies identified CRISPRs as central elements used by bacteria to protect against foreign nucleic acids; however, emerging evidence points to CRISPR involvement in bacterial virulence. The present study aimed to identify the participation of one CRISPR-Cas protein, Cas3, in the virulence of the oral pathogen Porphyromonas gingivalis, an organism highly associated with periodontitis. Our results show that compared to the wild type, a mutant with a deletion of the Cas3 gene, an essential nuclease part of the class 1 type I CRISPR-Cas system, increased the virulence of P. gingivalis. *In vitro* infection modeling revealed only mildly enhanced production of proinflammatory cytokines by THP-1 cells when infected with the mutant strain. Dual transcriptome sequencing (RNA-seq) analysis of infected THP-1 cells showed an increase in expression of genes associated with pathogenesis in response to Δ*cas3* mutant infection, with the target of Cas3 activities in neutrophil chemotaxis and gene silencing. The importance of *cas*3 in controlling virulence was corroborated in a Galleria mellonella infection model, where the presence of the Δ*cas*3 mutant resulted in a statistically significant increase in mortality of G. mellonella. A time-series analysis of transcription patterning during infection showed that G. mellonella elicited very different immune responses to the wild-type and the Δ*cas*3 mutant strains and revealed a rearrangement of association in coexpression networks. Together, these observations show for the first time that Cas3 plays a significant role in regulating the virulence of P. gingivalis.

**IMPORTANCE** Porphyromonas gingivalis is a key pathogen of periodontitis, a polymicrobial disease characterized by a chronic inflammation that destroys the tissues supporting the teeth. Thus, understanding the virulence potential of P. gingivalis is essential to maintaining a healthy oral microbiome. In nonoral organisms, CRISPR-Cas systems have been shown to modulate a variety of microbial processes, including protection from exogenous nucleic acids, and, more recently, have been implicated in bacterial virulence. Previously, our clinical findings identified activation of the CRISPR-Cas system in patient samples at the transition to disease; however, the mechanism of contribution to disease remained unknown. The importance of the present study resides in that it is becoming increasingly clear that CRISPR-associated proteins have broader functions than initially thought and that those functions now include their role in the virulence of periodontal pathogens. Studying a P. gingivalis *cas*3 mutant, we demonstrate that at least one of the CRISPR-Cas systems is involved in the regulation of virulence during infection.

## Abstract (precis)

Porphyromonas gingivalis is a key pathogen of periodontitis, a polymicrobial disease characterized by a chronic inflammation that destroys the tissues supporting the teeth. Thus, understanding the virulence potential of P. gingivalis is essential to maintaining a healthy oral microbiome. In nonoral organisms, CRISPR-Cas systems have been shown to modulate a variety of microbial processes, including protection from exogenous nucleic acids, and, more recently, have been implicated in bacterial virulence. Previously, our clinical findings identified activation of the CRISPR-Cas system in patient samples at the transition to disease; however, the mechanism of contribution to disease remained unknown. The importance of the present study resides in that it is becoming increasingly clear that CRISPR-associated proteins have broader functions than initially thought and that those functions now include their role in the virulence of periodontal pathogens. Studying a P. gingivalis *cas*3 mutant, we demonstrate that at least one of the CRISPR-Cas systems is involved in the regulation of virulence during infection.

## INTRODUCTION

Clustered regularly interspaced short palindromic repeat (CRISPR)-Cas systems provide prokaryotic cells with adaptive and heritable immunity. At present, there are two classes of CRISPR-Cas systems, with three types each, identified in bacteria (1). Structurally, a CRISPR-Cas genetic element consists of an array of repeats interspaced with relatively short DNA stretches, called spacers, with a set of *cas* genes nearby. The role of CRISPR-Cas in protection against bacteriophages and mobile genetic elements is well established (2, 3); however, mounting evidence indicates that CRISPR systems modulate a wide range of other biological processes of bacteria, including dormancy (4) and stress (5) as well as bacterial virulence and evasion of the immune system (6–9). The intracellular pathogen Francisella novicida uses a type II CRISPR-Cas system to evade innate immune detection by the host (7). Pseudomonas aeruginosa uses a type I CRISPR-Cas system, similar to one present in Porphyromonas gingivalis, to target the mRNA of the *las*R quorum-sensing gene to dampen recognition by the host (8). These novel findings point to a more comprehensive role for CRISPR systems in bacterial physiology and disease; however, the molecular mechanisms by which CRISPR-Cas systems contribute to such processes remain largely unknown (6, 9).

P. gingivalis is a natural member of the oral microbiome and a significant pathogen of periodontitis. It is hypothesized that the transition from periodontal health to disease coincides with the proliferation of this organism to high cell numbers in periodontal lesions, leading to tissue destruction due to its arsenal of specialized virulence factors (10, 11). Recent studies have also suggested a link between P. gingivalis and the risk for certain systemic conditions such as rheumatoid arthritis and cardiovascular disease (12, 13).

CRISPR-Cas systems have been identified in 19 genomes of P. gingivalis (14–16). Burmistrz et al. performed a functional analysis of P. gingivalis CRISPR-Cas systems and showed that all four CRISPR regions were transcribed and that at least one is active against dsDNA *in vivo* (17). Phillips et al. identified highly expressed transcripts of CRISPR regulatory small RNA (sRNA) *trans*-encoded in the region upstream of CRISPR-associated gene arrays in response to hemin limitation (18). Interestingly, one strain of P. gingivalis (JCVI SC001) was found to lack a CRISPR-Cas system and was isolated from a hospital sink drain (19).

The first indication that P. gingivalis CRISPR-associated proteins are important in virulence came from a previous study that we performed to define the metatranscriptome of periodontal disease progression (20). In that study, upregulation of all CRISPR-associated genes and, in particular, of *cas*3 in the systems present in P. gingivalis as well as in Tannerella forsythia, another organism associated with periodontitis, was observed (21); however, matching site comparisons from the patients that did not progress during this period showed no upregulation of CRISPR-associated genes (20). The *cas*3 gene is essential for CRISPR target interference in type I systems and encodes the nuclease that mediates target cleavage (22). Thus, this nuclease represents a target that is vital to understand the class 1 type I system for P. gingivalis virulence and disease. On the basis of this clinical information, in the present study, we performed targeted deletion of *cas*3 to begin to mechanistically understand type I CRISPR-Cas systems in P. gingivalis virulence capabilities. Using dual transcriptomic evaluations, coupled with cell and infection assays, we demonstrate that *cas*3 controls P. gingivalis virulence upon intracellular infection but seems to have no role in virulence when growing in the planktonic phase.

## RESULTS

### Deletion of *cas*3 does not affect growth or intracellular survival.

**FIG 1:**
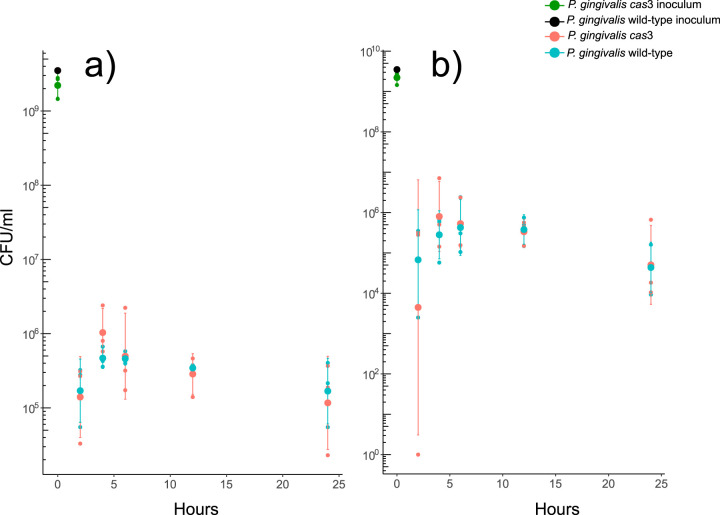
Intracellular and cell-associated P. gingivalis ATCC 33277 survival. THP-1 cells were infected with P. gingivalis ATCC 33277 wild-type and P. gingivalis ATCC 33277 Δ*cas*3 at the same levels of CFU/ml, and counts of CFU/ml were followed by a period of 24 h. Counts were obtained for intracellular P. gingivalis cells and for attached external cell-associated cells plus intracellular levels recovered after washing. (a) Intracellular surviving P. gingivalis in antibiotic-treated cells. (b) Cell-associated plus intracellular P. gingivalis in non-antibiotic-treated cells. All experiments were performed in triplicate.

Metatranscriptome results from our previous clinical study showed that P. gingivalis *cas*3 gene expression was 17-fold higher in those oral sites that progressed from clinically healthy to clinically diseased (see Fig. S1a in the supplemental material). As our clinical study could not distinguish between expression of *cas* genes from P. gingivalis living intracellularly and living extracellularly, we assessed the effect of mutating the *cas*3 gene on P. gingivalis growth rate extracellularly and intracellularly. To determine if *cas*3 is expressed during intracellular growth, we infected THP-1 macrophage-like cells with wild-type P. gingivalis. We then performed real-time PCR (RT-PCR) analysis on intracellular bacteria at 2 and 6 h after the monolayer's inoculation. In parallel, we also assessed the expression of the gene in planktonic growth in a serum-based medium. We observed an 8-fold increase in expression of the gene after 6 h of incubation in the intracellular bacteria, while no increase in expression was observed in planktonic growth after 2 and 6 h of incubation (Fig. S1b). Next, we assessed whether there were changes in the growth of the wild-type and mutant in planktonic growth and intracellularly in THP-1 cells. Our results showed no significant differences in either planktonic growth (Fig. S2a) or intracellular growth and no changes in the persistence of either P. gingivalis genotype in the two environments (Fig. 1). In our analysis, we distinguished between intracellular P. gingivalis cells obtained after antibiotic treatment and the fraction of P. gingivalis attached to the surface of THP-1 (cell-associated) plus intracellular P. gingivalis obtained after cell washing with no antibiotic treatment. We observed no effect of *cas*3 deletion on growth rates of P. gingivalis under any of those conditions (Fig. 1).

10.1128/mSystems.00852-20.1 FIG S1 (a) Organization of CRISPR-Cas system in P. gingivalis and results of the metatranscriptome analysis performed by Yost et al. (20). CRISPR-associated genes significantly upregulated during periodontal disease progression are indicated in red. The fold change values for the upregulated genes are indicated in parentheses. The spacers are indicated in purple. (b) Intracellular P. gingivalis increases the expression of *cas*3. THP-1 macrophage-like cells were cultured with P. gingivalis for 2 and 6 h, and RT-qPCR was used to measure the intracellular and extracellular bacterial expression of *cas*3. Extracellular bacteria (collected by washing) and intracellular bacteria (resistant to antimicrobial killing assay) were analyzed separately, and data were normalized to P. gingivalis cultivated in cell culture medium only. Download FIG S1, PDF file, 0.1 MB. Copyright © 2020 Solbiati et al. 2020 Solbiati et al. This content is distributed under the terms of the Creative Commons Attribution 4.0 International license.

10.1128/mSystems.00852-20.2 FIG S2 (a) Growth curves of wild-type and mutant strains growing on a human serum-based medium. (b) Venn diagram comparing differentially expressed genes upregulated in intracellular and planktonic growth. (c) Venn diagram comparing differentially expressed genes downregulated in intracellular and planktonic growth. Download FIG S2, PDF file, 0.2 MB. Copyright © 2020 Solbiati et al. 2020 Solbiati et al. This content is distributed under the terms of the Creative Commons Attribution 4.0 International license.

### Deletion of *cas*3 leads to significant changes in expression profiles of P. gingivalis when intracellular in THP-1 cells and an increase in activities associated with pathogenesis.

To begin determining the potential role of the Cas3 protein in the overall metabolism of P. gingivalis, we performed a transcriptome analysis of intracellular bacteria in THP-1 cells and in a human serum-based medium broth. The number of sequences in the THP-1 infection and planktonic growth experiments ranged from 49,592,338 to 82,270,371 sequences.

**FIG 2:**
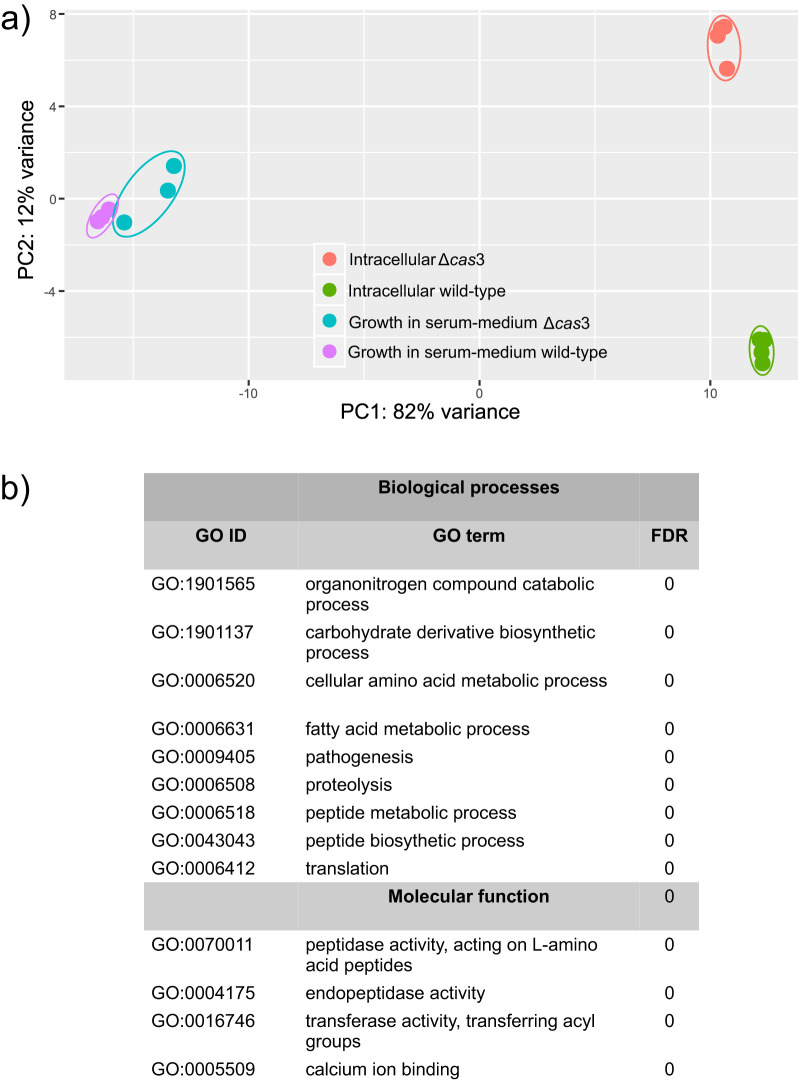
Effect of the *cas*3 deletion on gene expression of P. gingivalis extracellularly and intracellularly. Extracellular growth data refer to growth on a serum-based liquid medium. Intracellular growth data refer to growth in THP-1 cells. (a) Principal-component analysis (PCA) of the transcriptomes of the wild-type and Δ*cas*3 mutant strains in planktonic and intracellular growth. The transcriptomes from the different biological replicates for each condition are circled. (b) Biological processes and molecular function gene set enrichment analysis (GSEA) of gene ontology (GO) terms of P. gingivalis. Only P. gingivalis growing intracellularly showed differences in the GSEA results. FDR value, <0.01.

P. gingivalis growing in the serum-containing medium did not show any significant differences in gene expression profiles between wild-type and mutant strains. Principal-component analysis (PCA) of the complete profiles of gene expression showed similar patterns for the wild-type strain and the mutant (Fig. 2). In contrast to the results observed in planktonic growth, the patterns of the two strains growing intracellularly were very different (Fig. 2), suggesting that *cas*3 controls certain aspects of the metabolism of P. gingivalis when the bacterium is residing inside a host cell but has little to no significant effect when the organism is growing planktonically. More importantly, PCA showed that the expression profiles of the wild-type and mutant strains, growing intracellularly, clustered tightly based on the strain of origin but were very different between strains (Fig. 2) (see Table S1 in the supplemental material).

10.1128/mSystems.00852-20.9 TABLE S1 Differentially expressed genes in P. gingivalis in both planktonic and intracellular growth in THP-1 cells. Human genes that were differentially expressed in the THP-1 infection experiment are indicated. Download Table S1, XLSX file, 0.2 MB. Copyright © 2020 Solbiati et al. 2020 Solbiati et al. This content is distributed under the terms of the Creative Commons Attribution 4.0 International license.

Comparisons of the wild-type transcriptome to the mutant transcriptome in P. gingivalis in the planktonic and THP-1 infection experiments showed differential expression of many genes: 150 differentially expressed genes in the planktonic experiment and 814 in the THP-1 infection experiments (Table S1). Among the most highly upregulated genes in the Δ*cas*3 mutant in the THP-1 infection experiments, we found the genes for rubrerythrin, which plays a role in the oxidative stress response of P. gingivalis (23); for an ATPase; for an XRE family transcriptional regulator; and for a hypothetical protein. Interestingly, several Cas-associated protein genes (*cas*6, *cas*1, *cas*2, and *cmr*2) were found to be upregulated and a large number of mobile element proteins, 56 in total, were also upregulated in the *cas*3 mutant compared to the wild-type strain, among them a large number of loci of transposase in intracellular serine protease g1 (ISPg1), whose expression is induced when P. gingivalis is treated with H_2_O_2_ (24) (Table S1). Additional genes that have been associated with responses to oxidative stress and iron acquisition in P. gingivalis were also upregulated, including those encoding ferritin (25, 26), DNA-binding protein from starved cells (Dps) (25), ferredoxin (25), *vim*A (virulence modulating gene A) (27), putative universal stress protein UspA (28), hemagglutinin protein HagA (29), heme-binding protein FetB (29), and “upregulated in stationary phase protein A” (30).

Two genes with notably elevated expression were linked to the following proteins involved in modulation of the immune response: fig|431947.7.peg.735, which has been described as a gingipain-sensitive ligand A protein (GslA) (31), and the immunoreactive 46-kDa antigen fig|431947.7.peg.1742. Gingipain RgpA (fig|431947.7.peg.1936), an essential virulence factor in P. gingivalis, was significantly upregulated in the *cas*3 mutant. Downregulated genes in the *cas*3 mutant included the Tra protein genes of the conjugative transposons (*tra*E, *tra*F, *tra*I, *tra*J, *tra*K, *tra*L, *tra*N, *tra*O, *and tra*Q) and a large number of genes encoding ribosomal proteins both from the large subunit of the ribosome (LSU) and from the small subunit of the ribosome (SSU) (Table S1).

Gene set enrichment analysis (GSEA) of the differentially expressed genes showed that enriched biological processes were linked to pathogenesis and proteolytic activities in the P. gingivalis Δ*cas*3 mutant under conditions of growth inside the THP-1 cells (Fig. 2b, top panel). The same analysis showed enrichment of molecular functions associated with peptidase activity and binding to metal ions (Fig. 2b, bottom panel).

### The *cas*3 mutant induced changes in gene expression of THP-1 cells associated with immune response.

In parallel, we performed transcriptome analysis on the THP-1 cells infected with the two strains of P. gingivalis. PCA showed no apparent clustering of the expression profiles based on whether the THP-1 cells were infected with the wild-type or the mutant strain (Fig. S3a). We found that 2,526 genes were differentially expressed in comparisons of THP-1 cells infected with the wild-type and Δ*cas*3 mutant strains (Table S1). Intriguingly, the most abundant differentially expressed transcripts, all of them upregulated, were either microRNAs (miRNAs) or novel transcripts of unknown function (Table S1). A large number of the microRNAs identified in our study were previously linked to periodontal disease, including microRNA 130b, microRNA 19a, microRNA 20a, microRNA 27a, microRNA 302b, microRNA 30e, microRNA 374a, microRNA 374b, microRNA 593, microRNA 29b-1, and microRNA 21 (32, 33).

10.1128/mSystems.00852-20.3 FIG S3 (a) PCA of the expression profiles based on whether THP-1 cells were infected with the wild-type strain or the Δ*cas*3 mutant strain. (b) Enrichment of gene ontology terms for THP-1 cells infected with P. gingivalis. THP-1 cell genes were subjected to GOrilla analysis. The analysis shows results for GO terms corresponding to molecular functions. Download FIG S3, PDF file, 0.07 MB. Copyright © 2020 Solbiati et al. 2020 Solbiati et al. This content is distributed under the terms of the Creative Commons Attribution 4.0 International license.

**FIG 3:**
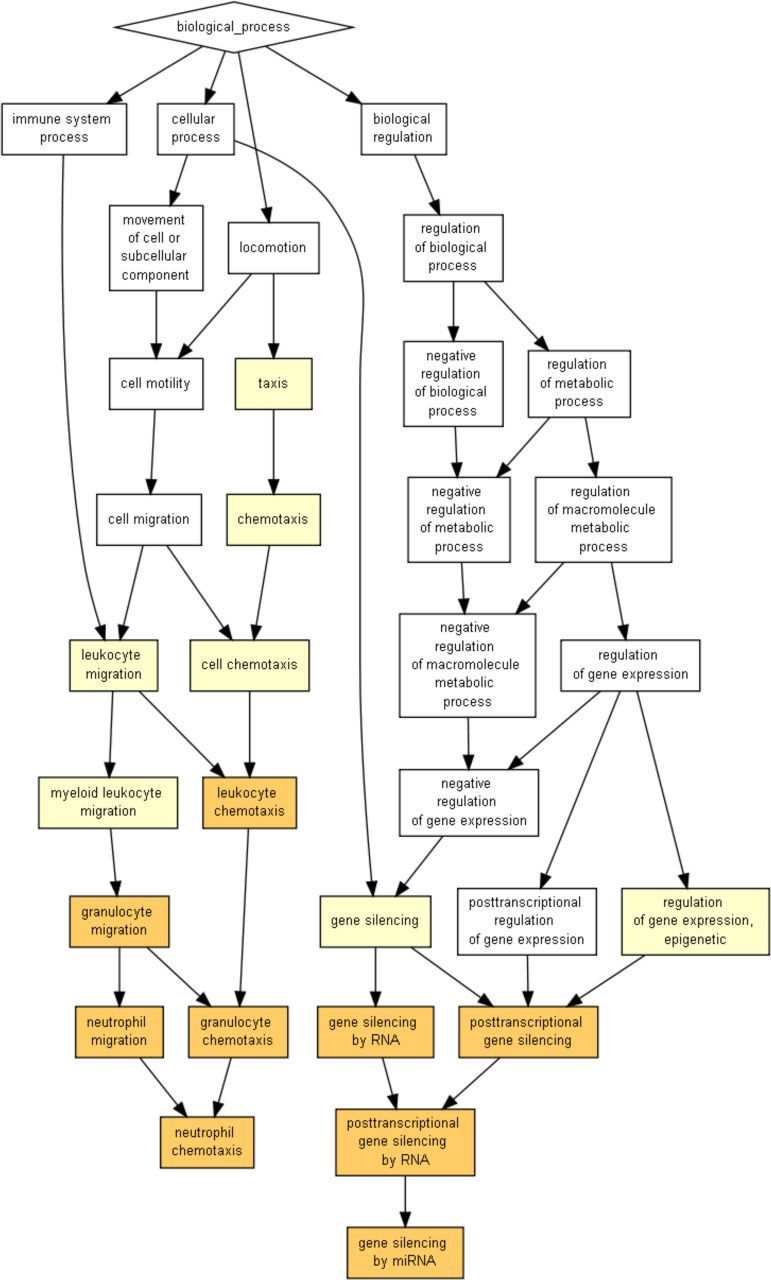
Enrichment of gene ontology terms for THP-1 cells infected with P. gingivalis. GOrilla analysis of THP-1 cell genes was performed. The data show results for GO terms of biological processes. The color indicates the degree of enrichment, from dark orange (significantly enriched) to white (not enriched).

The upregulated genes were mainly associated with cytoskeleton organization (actin-related proteins and ankyrin), cell death (BCL2 interacting proteins, caspase 9, and CASP8-like apoptosis regulators), and immune response (chemokine ligands and receptors; NF-κB inhibitors; interferon alpha and gamma receptors; interleukin 1 beta [IL-1β] and interleukin 1, 10, and 9 receptors; TEC proteins involved in intracellular signaling mechanisms of cytokine receptors; Toll-like receptor [TLR-2], TLR-4, and TLR-8; tetrahydrofuran [THF] alpha receptor; and tumor necrosis factor alpha [TNF-α]). Table S1 shows fold changes of the specific differentially expressed genes. Only one gene, the RNA5S9 gene, was found to have been downregulated when the cells were infected with the Δ*cas*3 mutant strain (Table S1). When we performed gene set enrichment analysis (GSEA) on Gene Ontology (GO) terms for those genes, we observed enrichment in genes associated with the immune response. Enriched biological processes were associated with neutrophil and leukocyte chemotaxis and also with gene silencing (Fig. 3), while enriched molecular functions were again found to be associated with gene silencing and chemokine and cytokine activities (Fig. S3b).

### Δ*cas*3 mutant had a modest effect on proinflammatory cytokine and chemokine production in the initial immune response of THP-1 to P. gingivalis.

**FIG 4:**
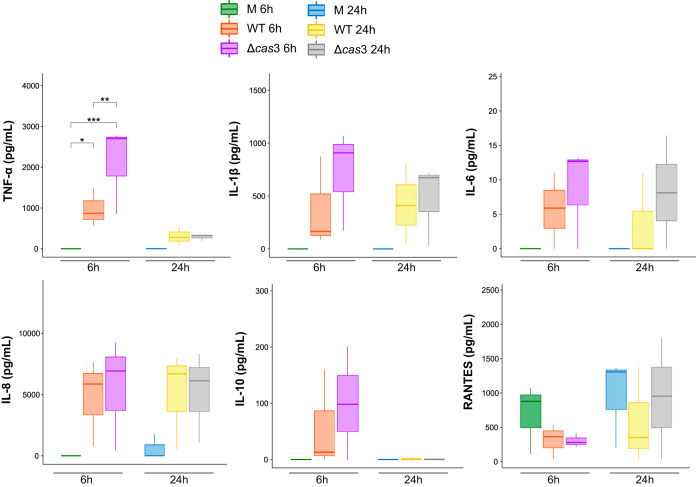
Influence on soluble immune mediators. After 6 and 24 h of incubation with the P. gingivalis wild-type strain (WT) or Δ*cas*3 mutant (Δ*cas*3), at a multiplicity of infection of 100, supernatant fluids from THP-1 cell culture were analyzed. Levels of TNF-α, IL-1β, IL-6, IL-8, IL-10, and RANTES were measured by multiplex immunoassay. M, medium only (unchallenged control). Data are presented as means ± standard errors of the means (SEM) (*n* = 3 independent experiments). TNF-α levels measured at 6 h showed significant differences in the two-way ANOVA. *, adjusted *P* = 0.032; **, adjusted *P* = 0.0228; ***, adjusted *P* = 0.00061.

As inflammation is a crucial driver of the bacterium-elicited tissue destruction that characterizes periodontal disease, we assessed the effect of mutating *cas*3 on the cytokine profiles of THP-1 macrophage-like cells. Using Luminex multiplex immunoassay, we measured cell culture supernatant fluid levels of the cytokines TNF-α, IL-1β, IL-6, IL-10, RANTES, and IL-8 at 6 h and 24 h following infection. Overall, we observed a slight increase in the levels of IL-1β, IL-6, and IL-10 in cells infected with the *cas*3 mutant compared to those infected with the wild-type strain, especially at 6 h, while RANTES levels showed decreases compared to the control, suggesting a potential inhibitory effect (Fig. 4). TNF-α expression levels were significantly higher in the mutant-infected cells than in the wild-type-infected cells (Fig. 4). The differences in the levels of the cytokines that we evaluated did not reach statistical significance. Intriguingly, the expression of *cas*3 intracellularly had its peak at 6 h as shown by quantification of the expression by real-time quantitative PCR (RT-qPCR) (Fig. S1b).

### Deletion of *cas*3 increases bacterial virulence of P. gingivalis ATCC 33277 in the Galleria mellonella killing assay.

**FIG 5:**
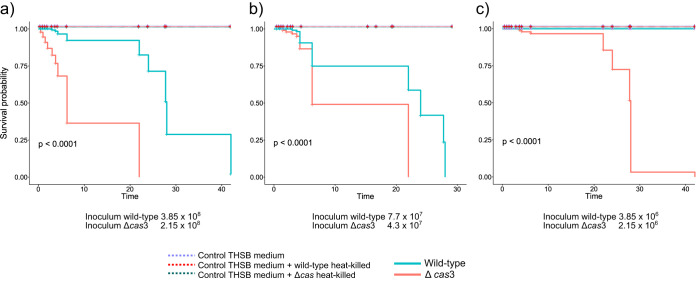
Survival curves in Galleria mellonella. Kaplan-Meier survival curves were determined. G. mellonella larvae were injected with the P. gingivalis ATCC 33277 wild-type and Δ*cas*3 strains. Three different dilutions of the inocula were tested. (a) Inoculum per worm in the wild-type strain, 3.85 × 10^8^ CFU; in the Δ*cas*3 mutant, 2.15 × 10^8^ CFU. (b) Inoculum per worm in the wild-type strain, 7.7 × 10^7^ CFU; in the Δ*cas*3 mutant, 4.3 × 10^7^ CFU. (c) Inoculum per worm in the wild-type strain, 3.85 × 10^6^ CFU; in the Δ*cas*3 mutant, 2.15 × 10^6^ CFU. Survival was monitored for 48 h. Larvae were also inoculated with 3 different negative controls: THSB medium where bacteria grew, THSB medium plus the wild-type strain (heat killed), and THSB medium plus the mutant strain (heat killed).

To directly assess the contribution of *cas*3 in virulence of P. gingivalis, we challenged groups of 15 G. mellonella larvae by injection with different dilutions (from 10^6^ to 10^8^ CFU) of the different strains. We found significantly higher mortality (*P <* 0.0001) for the groups of worms infected with the Δ*cas*3 mutant, with 100% mortality within the first 24 h when larvae were injected with 2.15 × 10^8^ CFU (Fig. 5a) and 90% mortality after 40 h when larvae were injected with 2.15 × 10^7^ CFU of the *cas*3 mutant (Fig. 5c). In contrast, only 20% of the larvae infected with the P. gingivalis wild-type strain died in the same period when larvae were injected with 3.85 × 10^8^ CFU (Fig. 5a), and larvae died after 40 h when injected with 3.85 × 10^6^ CFU of the wild-type strain (Fig. 5c). No larva mortality was observed in the control group until 3 days after inoculation (Fig. 5).

### Time-series dual-transcriptome results show that the transient response of G. mellonella larvae infected with the P. gingivalis wild-type strain is very different from the response seen with those infected with the Δ*cas*3 mutant.

To assess the “infection transcriptome” of wild-type P. gingivalis and the Δ*cas*3 mutant in the G. mellonella infection model, we performed a time-series dual-transcriptome analysis, selecting infected G. mellonella larvae at 0, 1, 2, and 6 h. We decided to use G. mellonella as a model because a strong correlation has been observed in microbial pathogenicity in G. mellonella and mammalian systems (34, 35) and because such an approach has been widely used as an initial screening model to assess virulence in a large number of different pathogens, including oral pathogens (36–38). The number of sequences in the G. mellonella infection experiments ranged between 74,281,712 and 86,843,647.

**FIG 6:**
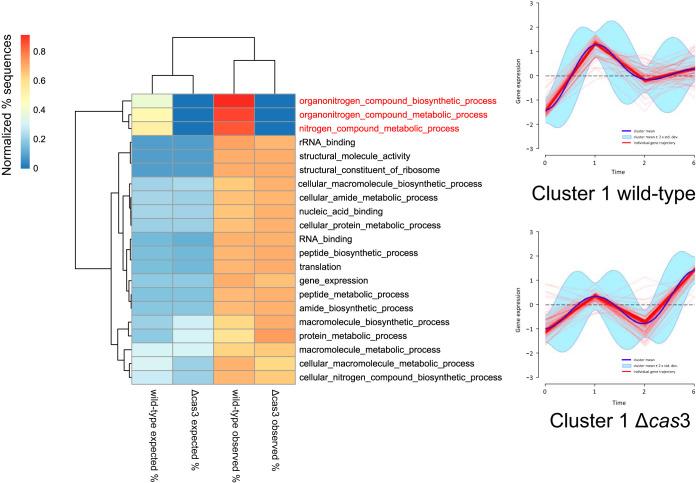
Heat map of GSEA of biological processes in Gene Ontology (GO) of P. gingivalis differentially expressed genes in cluster 1. DPGP analysis identified 6 clusters for the differentially expressed genes in the wild-type strain and 5 in the mutant. The more significant of the clusters (cluster 1) shared 103 differentially expressed genes and are represented by the clusters shown in the figure. Heat map data are clustered vertically based on the normalized frequency of expected or observed sequences and are horizontally arranged based on biological processes. The analysis produces two sets of results, the expected frequencies of sequences (from the reference set) and the observed frequencies of sequences (from the test set). Significance was assessed by Fisher’s exact test with an FDR value of <0.05. Test set, list of differentially expressed genes; reference set, list of all genes in the genome of P. gingivalis.

We identified 282 differentially expressed genes when we compared the infection transcriptomes of P. gingivalis wild-type strain and Δ*cas*3 mutant infecting G. mellonella (Table S2). Dirichlet process Gaussian process mixture model (DPGP) analysis identified 6 clusters for the differentially expressed genes in the wild-type strain and 5 in the mutant. The cluster with the larger number of genes included 103 genes differentially expressed in the wild-type strain and mutant (Table S2). Both strains showed increased activity in the first hour, but the wild-type strain showed a steeper upregulation (Fig. 6). Between the first and second hours, both showed a trend of decreased gene expression, and finally, after the second hour, another upregulation which, this time, was more pronounced in the mutant (Fig. 6). Enrichment analysis of these genes identified activities associated with nucleic acid binding, nitrogen metabolism, and translation (Fig. 6). The remaining clusters did not share any significant fraction of genes. However, when we associated those genes with their correspondent GO terms, aminopeptidase activity was found to have decreased in the mutant but not in the wild type and, interestingly, copper-exporting ATPase activity was found to have increased in the wild type whereas there was a peak at 2 h and a steep drop after that in the mutant (Fig. S4).

10.1128/mSystems.00852-20.4 FIG S4 Time-series clusters of P. gingivalis differentially expressed genes in infecting G. mellonella. DPGP analysis identified 6 clusters for the differentially expressed genes in the wild-type strain and 5 in the mutant. The clusters shown in the figure do not share any significant fraction of genes. Next to the figures are the molecular function GO terms associated with the genes in the cluster. GO terms were summarized using REVIGO. Download FIG S4, PDF file, 0.2 MB. Copyright © 2020 Solbiati et al. 2020 Solbiati et al. This content is distributed under the terms of the Creative Commons Attribution 4.0 International license.

10.1128/mSystems.00852-20.10 TABLE S2 Results of time-series differential expression analysis (DESeq2 spline) and DPGP clustering in the G. mellonella infection experiments. Download Table S2, XLSX file, 0.6 MB. Copyright © 2020 Solbiati et al. 2020 Solbiati et al. This content is distributed under the terms of the Creative Commons Attribution 4.0 International license.

In the transcriptome analysis of G. mellonella during infection, we found 4,490 differentially expressed genes along the course of the experiment. DPGP analysis identified 10 clusters for the set of G. mellonella larvae infected with the wild type and 8 clusters for the group infected with the Δ*cas*3 mutant. We performed Fisher’s test enrichment analysis on the genes listed in the clusters to assess global activity changes. Only one cluster (cluster 1) in the wild-type strain and the mutant shared a considerable number of differentially expressed genes; 90% (3,477) of the differentially expressed genes were identical (Fig. S5). Cluster 1 was significantly depleted of genes that are associated with nucleic acid binding. In both cases, the GO term “immune system processes” was enriched as well. Genes corresponding to this GO term are associated with activities of the innate immune response, among them those encoding the following: inactivation of cytokines (membrane alanyl aminopeptidases-like, LOC113515582, LOC113515584, and LOC113517732), phagocytic processes in dendritic cells and macrophages (aminopeptidases N-like, LOC113515678, LOC113514207, and LOC113515696), IL-1 receptor signaling pathway (myeloid differentiation primary response protein MyD88, LOC113513443), Toll-like receptor 6 (LOC113512725), immune cell lineage commitment and maintenance (RNA-binding protein lark, transcript variant X2, LOC113513283, and LOC113516103), and antimicrobial peptide (AMP) production (virescein-like, LOC113509608). Interestingly, the differential expression of MyD88 and Toll-like receptor 6, the latter of which is known to heterodimerize with Toll-like receptor 2 (TLR2), may suggest activation of TLR2 in a MyD88-dependent manner. However, the expression profiles were very different; while the wild-type strain showed a drop in expression of these genes in the first 2 h (Fig. S5a), the mutant showed an increase in expression during the first hour followed by a drop (Fig. S5b). Interestingly, two genes (LOC113517945 and LOC113515116) involved in the synthesis of phenoloxidase (PO), a key enzyme in melanin biosynthesis during melanization (39, 40), were differentially expressed in both cluster 1 of the wild type and cluster 3 of the Δ*cas*3 mutant (Fig. S5).

10.1128/mSystems.00852-20.5 FIG S5 GSEA of Gene Ontology (GO) terms of G. mellonella differentially expressed genes in cluster 1. (a) Cluster 1 for the set of G. mellonella larvae infected with the wild-type strain. (b) Cluster 1 for the set of G. mellonella larvae infected with the Δ*cas*3 strain. (c) Venn diagram of differentially expressed genes in cluster 1. The figure shows enrichment of biological terms. Significance was assessed by Fisher's exact test with an FDR value of <0.05. Test set, list of differentially expressed genes; reference set, list of all genes in the genome of G. mellonella. An asterisk (*) indicates that the gene was significantly more abundant on the reference list. Download FIG S5, PDF file, 0.2 MB. Copyright © 2020 Solbiati et al. 2020 Solbiati et al. This content is distributed under the terms of the Creative Commons Attribution 4.0 International license.

**FIG 7:**
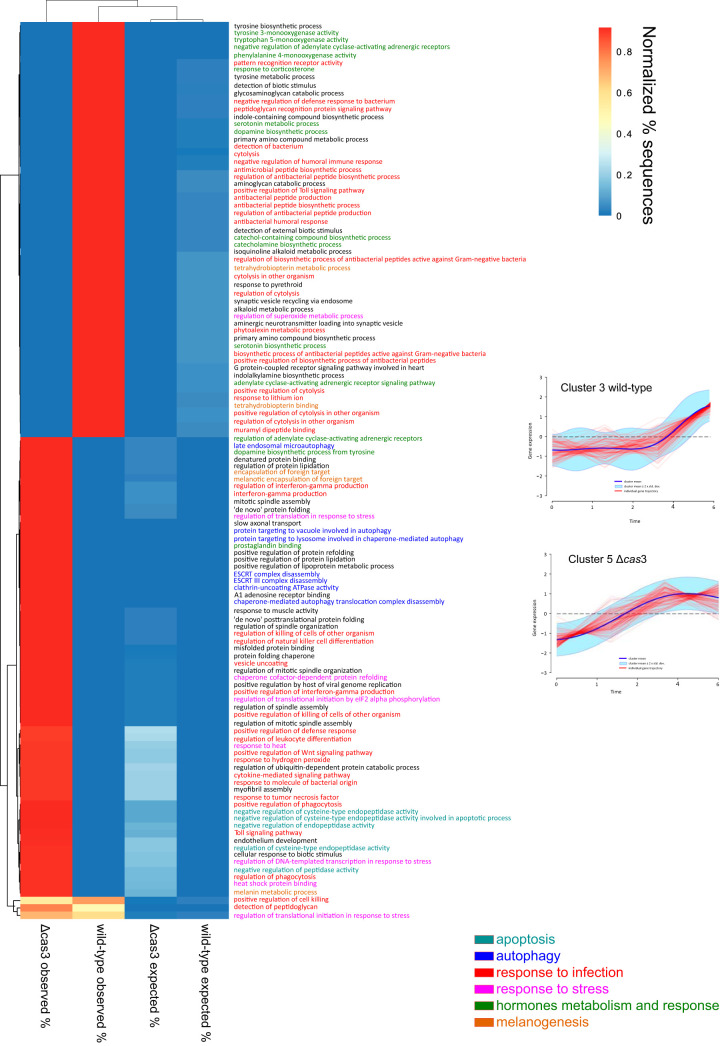
Heat map of GSEA of biological processes in Gene Ontology (GO) of G. mellonella differentially expressed genes in individual clusters. DPGP analysis identified 10 clusters for the set of G. mellonella infected with the wild-type strain and 8 clusters for the set infected with the Δ*cas*3 mutant. Two clusters, with very different profiles, showed similar activities: cluster 3 from G. mellonella infected with the wild-type strain and cluster 5 from G. mellonella infected with the Δ*cas*3 strain. Heat map data are clustered vertically based on the normalized frequency of expected or observed sequences and are horizontally arranged based on biological processes. The analysis produces two sets of results: the expected frequencies of sequences (from the reference set) and the observed frequencies of sequences (from the test set). Significance was assessed by Fisher’s exact test with an FDR value of <0.05. Test set, list of differentially expressed genes; reference set, list of all genes in the genome of G. mellonella.

The remaining clusters did not share a significant number of genes. Two clusters, with very different profiles, one from the group infected with the wild-type strain (Fig. 7, upper right panel) and one from the group infected with the Δ*cas*3 mutant (Fig. 7, lower right panel), showed similar activities. These activities were associated with apoptosis, autophagy, response to infection, response to stress, hormone metabolism, melanogenesis, and cytoskeleton organization (Fig. 7). Genes in wild-type cluster 3 maintained the same expression level for the first 2 h and did not show increased expression after that time (Fig. 7, upper right panel). In contrast, genes in cluster 3 in the Δ*cas*3 mutant showed increased expression levels immediately (Fig. 7, lower right panel). In both groups of worms, we observed activation of innate immune system genes in the Toll signaling pathway as well as activities associated with responses to stress, such as superoxide metabolic processes and response to hydrogen peroxide (Fig. S5; see also Fig. S6). Interestingly, the response of G. mellonella infected with the wild-type P. gingivalis strain seemed mediated primarily by the expression of AMPs (Fig. 7, upper right panel) and anionic antimicrobial peptide 2-like (LOC113519094), defensin (LOC113523425), defensin-like peptide (LOC113523442), gloverin (LOC113523269), and a lysozyme-like peptide (LOC113510919) (Table S2).

10.1128/mSystems.00852-20.6 FIG S6 GSEA of Gene Ontology (GO) terms of G. mellonella differentially expressed genes in individual clusters in wild-type strain infection. Clusters are indicated for the set of G. mellonella infected with the wild-type strain. The figure shows enrichment terms corresponding to biological processes. Download FIG S6, PDF file, 0.8 MB. Copyright © 2020 Solbiati et al. 2020 Solbiati et al. This content is distributed under the terms of the Creative Commons Attribution 4.0 International license.

In contrast, in the case of the group infected with the Δ*cas*3 mutant, we observed a response meditated by cytokines and phagocytosis (Fig. 7).

Additionally, while the G. mellonella group infected with the wild-type strain showed enrichment in a large number of activities associated with hormone metabolism (serotonin, catecholamine, corticosterone, and dopamine), that was not the case for the group infected with the Δ*cas*3 mutant (Fig. 7). On the other hand, apoptosis and autophagy were enriched in the group infected with the Δ*cas*3 mutant but not in the group infected with the wild-type strain (Fig. 7).

The last activity significantly altered during infection was cytoskeleton organization, with upregulation of genes associated with cytoskeleton organization in the wild-type group during the first hours of infection (Fig. S6, clusters 2 and 4). In contrast, the increase in activity in the Δ*cas*3 mutant group occurred latterly, and it was associated with repression of cytoskeleton turnover, such as negative regulation of actin filament depolymerization (Fig. S7, clusters 2 and 3).

10.1128/mSystems.00852-20.7 FIG S7 GSEA of GO terms of G. mellonella differentially expressed genes in individual clusters in the mutant strain infection. Clusters are indicated for the set of G. mellonella larvae infected with the Δ*cas*3 strain. The figure shows enrichment in terms corresponding to biological processes. Download FIG S7, PDF file, 0.7 MB. Copyright © 2020 Solbiati et al. 2020 Solbiati et al. This content is distributed under the terms of the Creative Commons Attribution 4.0 International license.

### Integrating dual transcriptomes of host-pathogen expression profiles in response to infection by coexpression network analysis.

Using the package ‘mixOmics,’ we performed coexpression analyses on the dual transcriptomes during infection of G. mellonella. ‘mixOmics’ allows us to distinguish between positive and negative correlations in the patterns of expression. As can be observed, heat maps corresponding to the correlations of the transcriptomes of the P. gingivalis wild-type strain (Fig. S8a) and the Δ*cas*3 mutant (Fig. S8b) with the host transcriptome reveal that the associations of gene expression of the two strains with the host are very different.

10.1128/mSystems.00852-20.8 FIG S8 Clustered image map of the discriminant features selected in the sPLS-DA. Genes of G. mellonella are indicated in the columns; genes of P. gingivalis are indicated in the rows. Positive correlations between the expression profiles of genes are indicated in red. Negative correlations between the expression profiles of genes are indicated in blue. (a) Correlations of expression profiles between the genes of the P. gingivalis wild-type strain and G. mellonella. (b) Relevance coexpression networks of negative correlations between genes of the P. gingivalis Δ*cas*3 mutant and G. mellonella. (c) Relevance coexpression networks of negative correlations between genes of the P. gingivalis wild-type strain and G. mellonella. (d) Coexpression network of the Δ*cas*3 mutant strain. (e) Venn diagram showing P. gingivalis genes with negative correlations to G. mellonella genes. Networks were obtained by stacked partial least-squares discriminant analysis (sPLS-DA) using the mixOmics package in R. Colors represent significantly enriched GO terms in the network of the G. mellonella transcriptome. Enrichment analysis and visualization of the results were performed using the R packages BiNGO and GOlorize. White nodes represent P. gingivalis genes. The associated GO names of the P. gingivalis genes in the network are indicated in red ovals. Download FIG S8, PDF file, 1.0 MB. Copyright © 2020 Solbiati et al. 2020 Solbiati et al. This content is distributed under the terms of the Creative Commons Attribution 4.0 International license.

As a result of our analysis, we obtained two coexpression networks, one for the wild type and one for the mutant. We further separated those correlation networks into positive-coexpression networks and negative-coexpression networks. Using these networks, we assessed both commonalities and differences that could indicate the different ways in which the two strains exerted their pathogenic effects in survival experiments. The first result that we observed was that the coexpression networks of negative associations in the wild-type strain and the mutant were completely different, with no overlap in structure. The analysis of differential networks did not result in any consensus network.

Moreover, although the P. gingivalis genes of those networks were associated with similar biological processes (cell redox homeostasis, lipid A synthesis, acetyl coenzyme A [acetyl-CoA] synthesis, and pathogenesis), the associated genes from the host were enriched in different activities. While the negative associations in the wild-type strain were mainly with membrane invagination and autophagic cell death in the host (Fig. S8c), the negative associations in the Δ*cas*3 mutant were mostly with positive regulation of apoptosis and innate immune response (Fig. S8d). Interestingly, although the GO terms associated with the P. gingivalis genes were similar, the genes were strain specific. Only 37% of the P. gingivalis genes in those coexpression networks were the same in the two strains (Fig. S8e).

**FIG 8:**
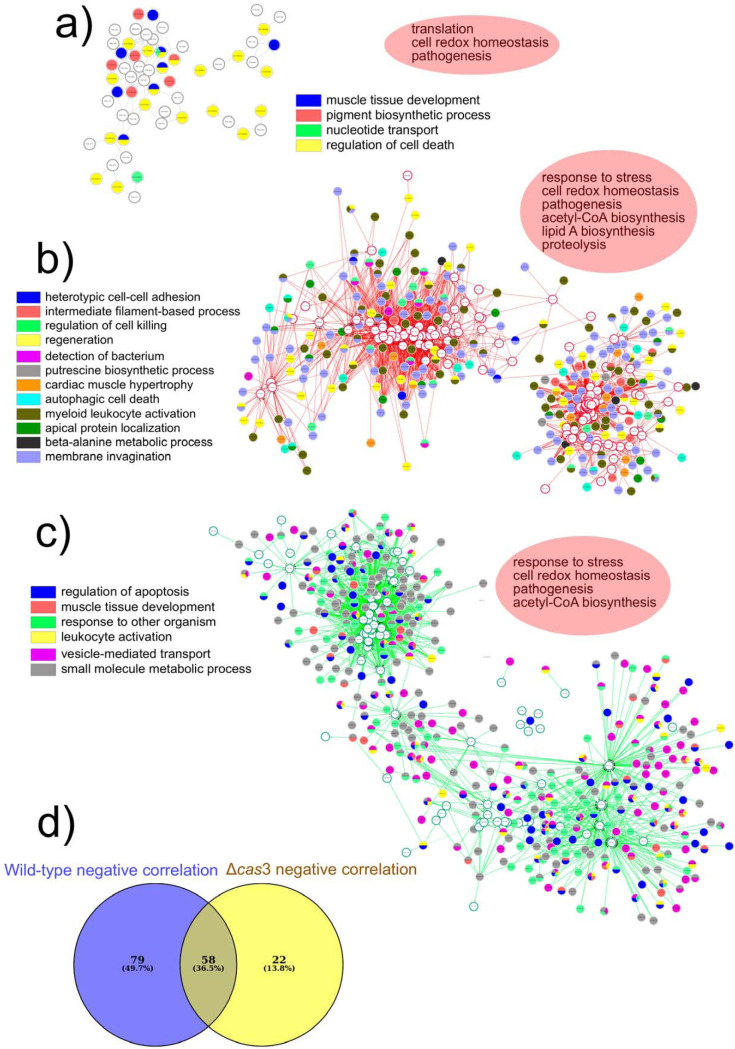
Relevance coexpression networks of positive correlations. Networks were obtained by sparse partial least-squares discriminant analysis (sPLS-DA) using the mixOmics package in R. Colors represent significantly enriched GO terms in the network of G. mellonella transcriptome. Enrichment analysis and visualization of the results were performed using the R packages BiNGO and GOlorize. In red ovals are the associated GO names of the P. gingivalis genes in the network. (a) Consensus coexpression network for both P. gingivalis strains. (b) A differential network where the connections were weaker or missing in the Δ*cas*3 mutant compared with the connections in the wild type. (c) A differential network where the connections were weaker or missing in the wild type compared with the connections in the Δ*cas*3 mutant. (d) Venn diagram showing P. gingivalis genes with negative correlations to G. mellonella genes.

The positive associations of coexpression were very different. Employing Diffany analysis, we found a consensus coexpression network in both the wild-type and mutant strains. This consensus network showed that expression profiles of genes associated with cell redox homeostasis and pathogenesis in P. gingivalis were positively correlated with the patterns of genes related to muscle tissue development, pigment synthesis, and cell death regulation in the host (Fig. 8a). Additionally, we identified differential networks, defined as networks with identical edges but with opposite significantly different correlations (41). The two differential networks are shown in Fig. 8. Panel b of Fig. 8 shows the differential network where the connections were weaker in the mutant than in the wild-type connections or were missing in the mutant connections. P. gingivalis genes in this network were associated with response to stress, cell redox homeostasis, acetyl-CoA synthesis, lipid A synthesis, proteolysis, and pathogenesis. These activities were positively correlated with genes in the host enriched in GO terms associated with membrane invagination, myeloid leukocyte activation, and regeneration (Fig. 8b). Conversely, for the differential network where the connections were weaker than or missing in the links in the wild type compared with the links in the *cas*3 mutant, P. gingivalis genes were similarly associated with response to stress, cell redox homeostasis, acetyl-CoA synthesis, and pathogenesis. However, the enrichment of host coexpressed genes was mainly related to the regulation of apoptosis, vesicle-mediated transport, and small-molecule metabolic processes (Fig. 8c). As in the case of the negative-coexpression networks, despite the similarities in the GO term activities of the P. gingivalis genes, they were somewhat different. Only 36% of the genes in those networks overlapped in the two strains (Fig. 8d).

## DISCUSSION

CRISPR-Cas systems are now known to modulate a variety of microbial processes; however, the contributions of these systems to the virulence of periodontal pathogens are thus far unknown. Our prior work focusing on the transition from oral health to disease showed that within individual patients, the majority of genes belonging to the CRISPR-Cas loci in P. gingivalis in sites during the transition to periodontal disease were highly upregulated compared to the sites in the same patients that did not progress, where CRISPR-associated gene expression did not change (20). In this article, we report that an in-frame mutant with a deletion of the *cas*3 gene, encoding an essential nuclease in type I-C systems, increased the virulence of the mutated strain, demonstrating that at least one of the CRISPR-Cas systems of P. gingivalis (type I-C system) is involved in regulating the virulence of P. gingivalis during infection.

The descriptions of mechanistic use of CRISPR-Cas systems by bacteria have focused mainly on its innate defense nature in protecting microbes from exogenous nucleic acids and phages (2). However, Lum et al. showed that although transcription of oral CRISPR loci is relatively ubiquitous, highly expressed CRISPR spacers do not necessarily target the most abundant oral phages (42). Moreover, recently, new functions, other than defense, have been attributed to CRISPR systems of the oral microbiome (43). Even though bacteriophages have been isolated from oral bacteria, a bacteriophage for P. gingivalis has yet to be isolated despite the efforts in this area (44, 45). Although the P. gingivalis CRISPR-Cas systems may defend against oral phages, that prior work and our clinical data do not support the notion that oral phages alone are responsible for the changed expression occurring mainly in the context of the transition from periodontal health to disease. CRISPR-Cas genes were highly upregulated at sites with clinical evidence of transition to disease compared to healthy sites in the same individual; it would be expected that under both conditions at both sites, oral phages would be present.

An expansion of our understanding of the importance of CRISPR-Cas systems to bacteria beyond protection from nucleic acid insult has come from emerging literature on their role in intracellular persistence of pathogens such as Campylobacter jejuni (6), Francisella novicida (7), Legionella pneumophila (46), Neisseria meningitidis (47), and Listeria monocytogenes (9). P. gingivalis, as well as several other oral microbiome members, is a regular component of the intracellular communities of oral epithelial cells (48, 49). Intracellular survival is an essential part of the P. gingivalis lifestyle in the periodontal pocket as well a key element in transmission from cell to cell in the epithelium, enabling avoidance of the humoral immune response (49–51). CRISP-Cas systems are ideal elements for regulating intracellular survival, given how quickly they can control gene expression in the short time available for cell invasion. Therefore, we hypothesized that CRISPR-Cas systems in P. gingivalis might be involved in intracellular survival. Interestingly, mining a data set from an independent study which examined the genome of P. gingivalis to identify genes essential for colonization of epithelial cells, we noted that all genes in the type I-C system locus, except for *cas*2 (fig|431947.7.peg.1924), were significantly associated with successful infection in that prior investigation (52). Here, focusing on *cas*3, our findings confirm this prior observation as we found that *cas*3 transcription was induced in P. gingivalis growing intracellularly but not planktonically.

Nonetheless, *cas*3 mutation did not alter the planktonic or intracellular growth of P. gingivalis compared to the wild type. A recent study on the effects of deleting *cas*3 in Salmonella enterica showed no differences in liquid growth. In contrast to our results, those researchers found a decrease in intracellular survival in the mutant (53). The expression profiles of wild-type P. gingivalis and the *cas*3 mutant were very similar when the bacteria were placed in human serum-based medium, but when they colonized the human macrophage-like THP-1 cell line, the transcriptome profiles of the wild-type and mutant strains were very different. The only activities that were altered under both sets of growing conditions were activities associated with adhesion, probably because fimbrial genes, including *fim*A, were downregulated in the mutant. In S. enterica, deletion of *cas*3 leads to downregulation of *saf*A, *saf*B, *saf*C, and *saf*D genes in the fimbrial operon (53). The mechanisms by which *cas*3 controls levels of transcription of fimbrial genes remain unknown. However, given the importance of fimbria in attachment of bacteria to host cells and the similarity of our findings of reduced transcription of fimbrial genes due to *cas*3 deletion to those observed with S. enterica, this mechanism of CRISPR control of infection should be investigated further.

Deletion of *cas*3 had a significant effect on the pathogenesis of intracellular P. gingivalis, as well as on the immune response of THP-1 cells. We observed an upregulation of genes associated with responses to oxidative stress and iron acquisition in the mutant. These activities are at the core of the mechanisms of pathogenicity in P. gingivalis (23–26, 30, 54, 55), and an increase in their levels increase virulences. Unexpectedly, we did not observe changed secretion of TNF-α and IL-1β or other proinflammatory cytokines or chemokines between the cells infected with the wild type and those infected with the mutant. However, a modest trend of increased inflammatory molecule production coincides in time with the peak in the number of intracellular transcripts for *cas*3. These results agreed with the transcriptome analysis, where IL-1β and TNF-α genes were upregulated in the cells infected with the mutant. In a C57BL/6J mouse model, Li et al. found that secretion of TNF-α and IL-1β after 2 h of infection was higher when mice were infected with a CRISPR-Cas P. aeruginosa mutant than when they were infected with the wild-type strain (8).

Furthermore, infection with the Δ*cas*3 mutant strain altered neutrophil chemotaxis and gene silencing by microRNA (miRNA). We observed a high number of microRNAs upregulated in the cells infected with the Δ*cas*3 mutant. Previous studies had also shown that during periodontitis, the expression of microRNAs in humans is altered (32, 33), a fraction of which we found differentially expressed in our study.

Although the number of studies is still limited, the dampening of the immune response seems to be an element common to the mechanisms by which CRISPR-Cas systems control virulence. F. novicida evades the innate immune response by regulating levels of the endogenous transcript of bacterial lipoprotein (BLP; FTN_1103) via a CRISPR/Cas-associated RNA (small Cajal body-specific RNA [scaRNA]) (7), and P. aeruginosa evades host defenses by targeting mRNA of the quorum-sensing regulator LasR to dampen the recognition by TLR-4 (8). Our results agreed with those two studies (7, 8) in that mutation of *cas*3 increased virulence. The exception to this is S. enterica, where deletion of *cas*3 weakened bacterial virulence (53). Interestingly, both P. aeruginosa and S. enterica possess type I CRISPR-Cas systems but target quorum-sensing systems, and yet different outcomes are seen (8, 53). P. gingivalis employs an AI-2/LuxS-mediated quorum-sensing system. We did not see any differences in the levels of expression of *lux*S in our experiments performed with THP-1 cells. However, in our time-series analysis of infection in G. mellonella, *lux*S was differentially expressed, with a peak in the first hour in the wild type and a peak at 6 h in the mutant. Note that P. gingivalis does not possess bacterial lipoprotein (BLP) or LasR homologs (data not shown). Thus, it would appear that neither BPL nor LasR represents common mechanisms of action for P. gingivalis Cas3, thus supporting the notion that another, unknown system is likely involved for at least this organism. Interestingly, 11 of the 1,188 P. gingivalis spacers identified by Watanabe et al. (56) had perfect matches with genes in P. gingivalis ATCC 33277. Most of those genes are hypothetical proteins, but we found that one of the targets upregulated in our analysis was a putative TonB gene, which may suggest a potential role in regulating iron metabolism by the CRISPR-Cas system in P. gingivalis.

The most direct evidence of the increase of virulence in the mutant strain came from our experiments using G. mellonella as an infection model. Previous work with several microbial pathogens demonstrated a positive correlation between this model’s results and results from other mammalian disease models (35, 57, 58). Innate immune responses of G. mellonella are comparable with vertebrate innate immune responses and involve recognition of the bacteria and production of antimicrobial molecules (59). Moreover, there is good evidence that the G. mellonella model is suitable for studying pathogenesis and immune responses in human oral pathogens (60), including P. gingivalis (38, 61). Our results showed a significant increase in virulence measured as the death rate of the infected larvae with the *cas*3 mutant during the 48 h of the experiment.

All previous studies on the role of CRISPR-Cas systems in virulence were performed at a single time point for comparisons of conditions. Time-series analysis of the G. mellonella model of infection gave us a dynamic picture of the process rather than just a snapshot of the differences in gene expression at a given time. The most striking results of those experiments are those demonstrating the great differences in the host’s infection transcriptomes. Activities associated with apoptosis, autophagy, response to infection, response to stress, hormone metabolism, melanogenesis, and cytoskeleton organization showed distinct time-dependent behavior. Larvae infected with the wild type maintain the same level for the first 2 h, and it is not until after that time that they increased their expression. In contrast, the Δ*cas*3 mutant increased the expression level of those activities immediately. In human coronary artery endothelial cells (HCAEC) and human aortic endothelial cells (HAEC), P. gingivalis activates cellular autophagy to provide a replicative niche while suppressing apoptosis (62, 63). The increase in autophagic activity in the mutant-infected larvae seems to indicate a faster infectious process. Moreover, P. gingivalis additionally induces endoplasmic reticulum (ER) stress-mediated apoptosis in human umbilical vein endothelial cells (HUVEC), followed by an autophagic response (64).

The immune response of G. mellonella consists of two tightly interconnected components, i.e., cellular and humoral components, that collaborate to ensure the best protection to the insects (65, 66). The cellular response is mediated by hemocytes and involves responses such as phagocytosis, encapsulation, and nodulation (67). The humoral response consists of the synthesis of defense molecules, including antimicrobial peptides and melanin, which are involved in encapsulating the invading pathogen and also stimulate the defense activity of other antimicrobial molecules (67).

We observed two distinct response profiles of the larvae depending on the infecting strain. The first pattern of response was seen with the G. mellonella group infected with the wild type and seemed driven primarily by a humoral response corresponding to the synthesis of AMPs. In periodontitis, neutrophils are recruited to sites of injury and secrete a rich mixture of AMPs. Over 45 AMPs have been identified in saliva and gingival crevicular fluid (GCF). Still, their biological function with respect to disease may be more complex than just the killing of bacteria since the levels of several AMPs are below the MIC for oral pathogens (68). Although they are effective against planktonic bacteria, effectiveness against biofilms is not apparent (69).

As a part of the immune response, G. mellonella synthesizes a wide variety of AMPs (67, 70–72). Additionally, the G. mellonella group infected with the wild-type strain showed enrichment in a large number of activities associated with hormone synthesis and response. This increase in hormone production is most likely linked to melanogenesis. Melanization is one of the primary defense reactions to the encapsulation of pathogens in G. mellonella (66). Bacterial infections induce bursts of dopamine that can be related to the direct participation of dopamine in the phenoloxidase cascade, a major defense system in many invertebrates, ultimately leading to melanization of pathogens and damaged tissues (73).

In contrast, defense against infection with the Δ*cas*3 mutant was primarily mediated by cellular responses, including synthesis of cytokines and phagocytosis, with enrichment in apoptosis and autophagy activities. The presence of IL-1α and TNF-α-like molecules in the hemocytes of G. mellonella (74) and transcriptome analysis of this organism identified homologs of a cytokine called Spätzle, which functions in the antimicrobial immune response in larvae and adults (75). In Drosophila melanogaster, Spätzle functions as a circulating cytokine and specifically binds with high affinity to Toll, thereby activating the Toll signaling pathway (76). G. mellonella possesses hemocytes that can phagocyte intruders or capture them in multicellular structures called nodules of capsules (67). Granulocytes, representing a type of hemocyte in G. mellonella, showed high levels of expression of the autophagy response through microtubule-associated LC3 when G. mellonella was infected with a high-virulence strain of Actinobacillus pleuropneumoniae, whereas treatment with a low-virulence strain induced lower levels of expression of this protein (36). Moreover, in Helicobacter pylori, the killing of larvae always correlated with the induction of apoptosis in hemocytes (37).

We also observe changes in cytoskeleton metabolism as part of the cellular defense against infection. Infection of P. aeruginosa affects G. mellonella hemocyte cytoskeleton rearrangement, disabling cellular immunity (77). Hemocyte motility relies on the continual restructuring of the cytoskeleton. In the wild-type-infected larvae, we observed upregulation of genes associated with cytoskeleton reorganization in the first hours of infection. Simultaneously, in the Δ*cas*3 mutant group, the increase in activity occurred later, and it was associated with the cytoskeletal organization’s inhibitory activities.

Although the two models that we used in our transcriptome infection experiments (human THP-1 cells and G. mellonella) and the experiment designs (cross-sectional data and time-series data) were very different, the transcriptome results suggest that the differential immune responses elicited by P. gingivalis wild-type and *cas*3 mutant strains in G. mellonella and THP-1 cells are somehow controlled by the CRISPR-Cas system, controlling nucleic acid binding (mRNA binding), migration of immune cells, and cytokine and chemokine responses, which are activities altered in the transcriptomes of both THP-1 cells and G. mellonella.

Most studies of infectious disease pathogenesis focus on either the host or the pathogen, even though their interaction determines the outcome. Moreover, such studies generally analyze transcriptomes at only a single stage of the infection process, missing the changes across different stages of the disease, which is a dynamic process. The presence of coexpression networks does not necessarily imply causality, but they are increasingly employed in bioinformatics applications to explore the system-level functionality of genes in host-pathogen interactions (78, 79). Analyses of our study’s relevant coexpression networks showed that the levels of expression of P. gingivalis genes associated with G. mellonella were similar with respect to the GO categories in wild-type and Δ*cas*3 mutant infections. However, the specific genes related to those GO categories did not overlap. We identified a consensus network response in G. mellonella that probably acts as a core response against both strains. Not surprisingly, it includes activities essential in the immune response of larvae against pathogens: pigment synthesis and regulation of cell death in the host (36, 66, 67, 80). To identify the specific differences in coexpression between the two strains, we generated differential coexpression networks, defined as networks with shared edges but with opposite significantly different correlations (41). The connections that were weaker or missing in the mutant compared with the connections in the wild-type activities were positively correlated with genes in the host enriched in GO terms associated with membrane invagination, myeloid leukocyte activation, and regeneration. G. mellonella does not possess leukocytes, and the GO terms are probably related to hemocyte activity. Conversely, the differential network where the connections were weaker or missing in the wild type compared with the connections in the mutant, enrichment of host coexpressed genes, was mainly associated with regulation of apoptosis, vesicle-mediated transport, and small-molecule metabolic processes.

In conclusion, we have demonstrated a direct link of the presence of *cas*3 gene with an increase in the virulence of P. gingivalis, as has been described previously for other pathogens (7, 8). In addition to revealing the importance of a CRISPR-Cas system in controlling the virulence of P. gingivalis, we found that the lack of *cas*3 had significant impacts leading to the rearrangement of the host’s response, mainly when evaluated in our time-series infection modeling. These findings extend and further support the notion that CRISPR-Cas systems can effectively reshape bacterial virulence and escape the host immune defense.

## MATERIALS AND METHODS

### Bacterial growth conditions.

Porphyromonas gingivalis ATCC 33277 was cultured anaerobically at 37°C. Cells were maintained on brain heart infusion (BHI)-blood agar plates supplemented with 5 μg/ml hemin and 1 μg/ml menadione (vitamin K). Two different liquid growth media were used in the experiments. For the construction of the mutant and the infection experiments, we used BHI broth supplemented with 1 mg/ml yeast extract, 5 μg/ml hemin, and 1 μg/ml menadione. For the transcriptome experiments, we grew P. gingivalis in 20% heat-inactivated human serum (Sigma-Aldrich H3667-20ML) supplemented with 5 μg/ml hemin and 1 μg/ml menadione as described previously by Grenier et al. (81).

### Construction of a CRISPR-Cas3 gene knockout strain of Porphyromonas gingivalis.

We constructed the fig|431947.7.peg.1929 (PATRIC annotation [82]) (see Fig. S1a in the supplemental material) gene knockout strain by replacing the whole gene with an erythromycin resistance cassette. First, we constructed a plasmid (pUC19) carrying the erythromycin resistance cassette (*erm*F gene from pVA2198) flanked by the 1-kb region upstream and downstream of fig|431947.7.peg.1929. The fig|431947.7.peg.1929 gene was replaced by the erythromycin cassette and was kept in frame with the rest of the genes in the operon. The construct was made in such a way that the complete fig|431947.7.peg.1929 gene was replaced by the erythromycin cassette and was kept in frame with the rest of the genes in the operon. This construct was made using a NEBuilder HiFi DNA assembly kit. The kit was used with 100 ng of P. gingivalis genomic DNA according to the manufacturer's protocol. The NEBuilder Assembly Tool (NEB) was used to design the primers for the NEBuilder HiFi DNA kit protocol. Dh5-alpha chemical competent cells were used for the transformation. The resulting plasmid-transformed Dh5-alpha cells were selected on ampicillin LB plates. The plasmid was extracted using EZNA plasmid DNA minikit II (Omega) and sequenced. After verification by sequencing, the plasmid was named pFLUF001.

Primers JS_Cas3KOPCR1-F (5′-GGAAGTGACCGTTATCGAAGAT-3′) and JS_Cas3KOPCR1-R (5′-GCCTTACGAATAGGCCATAAGA-3′) were used to amplify, from pFLUF001, the 2.7-kb DNA fragment containing the erythromycin cassette and its flanking regions. Pfu polymerase (Fermentas) was used according to the manufacturer’s protocol. The amplified fragment was cleaned using an EZNA gel extraction kit (Omega) and used for electroporation of P. gingivalis electrocompetent cells. P. gingivalis ATCC 33277 electrocompetent cells were made by growing the cells on tryptic soy broth (TSB) supplemented with hemin and vitamin K to an optical density at 600 nm (OD_600_) of 0.6 to 0.7. After centrifugation, the cells were washed twice in ice-cold electroporation buffer (10% glycerol, 1 mM MgCl_2_) and finally resuspended in a minimal amount of buffer. A 100-μl sample of P. gingivalis electrocompetent cells was electroporated with different amounts of the purified DNA fragment. Cells were plated on BHI-blood agar plates supplemented with hemin and vitamin K and 10 μg/ml of erythromycin. After anaerobic incubation at 37°C for 7 days, the resulting colonies were streaked on new plates, and single colonies were obtained. The fig|431947.7.peg.1929 (Cas3) gene knockout strain was verified by colony PCR using primers from the erythromycin cassette, and the adjacent region to the flanking region was amplified and cloned in pFLUF001. The amplified product was confirmed by sequencing. The correct gene knockout strain was grown on liquid media, and glycerol and dimethyl sulfoxide (DMSO) stocks were prepared and stored in an −80°C freezer.

### THP-1 cell culture.

As described previously (83), human monocyte/macrophage cell line THP-1 (ATCC, TIB-202) was cultured at 37°C in a 5% CO_2_ incubator in RPMI 1640 supplemented with 10% heat-inactivated fetal bovine serum, l-glutamine (2 mM), penicillin/streptomycin (100 U/100 μg/ml), HEPES (10 mM), sodium pyruvate (1 mM), glucose (4.5 mg/ml), sodium bicarbonate (1.5 mg/ml), and 2-mercaptoethanol (Sigma-Aldrich) (0.05 mM). Cells were adjusted to 5 × 10^5^ viable cells/ml, and, to induce differentiation into a macrophage-like state, THP-1 cells were placed into fresh medium containing 100 ng/ml phorbol 12-myristate 13-acetate (PMA; Sigma-Aldrich) and 1 ml of THP-1 cells was added to each well of 24-well cell culture plates. After 48 h of incubation, the cell culture medium was replaced by antibiotic-free medium, and cells were used in challenge studies.

### Bacterial infection experiments.

P. gingivalis wild-type and Δ*cas*3 mutant cells were harvested from the BHI broth culture by centrifugation, and the bacteria were washed three times in RPMI 1640 medium and adjusted to an OD_660_ of 1.0 (approximately 1 × 10^9^ CFU/ml) and added to PMA-activated THP-1 cells at a multiplicity of infection of 100, in the antibiotic-free medium, and after 2 h of infection, the THP-1 cells were washed to remove nonadherent bacteria. After 2 h of incubation, the THP-1 cells were treated for 1 h with metronidazole (200 μg/ml)/gentamicin (300 μg/ml) to kill extracellular bacteria (51), were washed to remove antibiotics, and then were lysed with sterile water, followed by addition of an equal volume of 2× phosphate-buffered saline (PBS). Serial 10-fold dilutions of each lysate were plated on blood agar plates. CFU/ml of P. gingivalis was calculated. In separate experiments, THP-1 cells were cultured with the P. gingivalis ATCC 33277 wild-type strain or the Δ*cas*3 mutant, and both cell culture supernatant fluids and RNA were harvested for measurement of cytokine expression and RNA was extracted for dual transcription. Differences in bacterial counts were assessed by performing a Kruskal-Wallis test corrected for multiple comparisons using the function ‘kruskal’ from the ‘agricolae’ package in R, with a false-discovery rate (FDR) value of <0.05.

### RNA extraction.

Total RNA was extracted from those samples using a *mir*Vana RNA isolation kit (Life Technologies). Samples were bead-beaten for 1 min at maximum speed with 300 μl of 0.1-mm diethyl pyrocarbonate (DEPC)-treated zirconia-silica beads (BioSpec Products) in the *mir*Vana lysis buffer. Bacterial rRNA was depleted using Ribo-Zero gold rRNA removal kits (Illumina) following the manufacturer's protocol. In the THP-1 infection experiments, sequencing of total RNA, including human and bacterial mRNA, was performed. Their identification was performed bioinformatically, as described below.

G. mellonella RNA was extracted using a *mir*Vana RNA extraction kit (Thermo Fisher Scientific) with minor modifications. Individual larvae were washed with autoclaved PBS and were cut at the distal end. The internal content was snap-frozen in liquid nitrogen, homogenized and pulverized, and immediately incubated at room temperature for 1 h in lysis buffer (*mir*Vana kit; Thermo Fisher Scientific). After the lysis steps, the manufacturer’s instructions were followed.

In the experiments performed for analysis of G. mellonella infection, we divided the total RNA into two subsamples. One half was used for transcriptome analysis of G. mellonella, for which we used a Dynabeads mRNA purification kit to isolate eukaryotic mRNA for transcriptome analysis. The other half was depleted of eukaryotic mRNA using a MICROBEnrich kit (Thermo Fisher Scientific, catalog no. AM1901).

### RT-qPCR quantification of *cas*3 transcripts.

From the initial RNA extraction, possible traces of DNA were removed using Ambion’s Turbo DNA-free kit (Ambion) following the manufacturer’s instructions. The volume of Turbo DNase I (Ambion’s Turbo DNA-free) was increased to 3 μl, and the reaction mixture was incubated at 37°C for 60 min. RNA (100 ng) was reverse transcribed with random hexamer primers and with SuperScript II reverse transcriptase (Invitrogen) following the manufacturer's instructions. Reverse transcription was performed at 42°C for 2 h, after an initial incubation step of 10 min at 25°C. The synthesized cDNA was used in a DyNAmo Flash SYBR green qPCR kit (New England Biolabs, Ipswich, MA) according to manufacturer’s instructions, using the specific primers for the genes of interest. To compare the relative expression levels of genes, we modified the threshold cycle (2^−ΔΔ^*^CT^*) method (84), and we used the formula cDNA_mutant_/cDNA_control_ = (1 + E_cDNAcontrol Only two_)^CtcDNAcontrol^/(1 + E _cDNAmutant_)^CtcDNAmutant^ to take into consideration the different amplification efficiencies in the separate qPCR runs.

### RNA-seq library construction.

Sequencing was performed at the Interdisciplinary Center for Biotechnology Research (ICBR) at the University of Florida using a HiSeq 2500 machine. First, rRNAs were removed from total RNA by the use of an Illumina Ribo-Zero gold rRNA removal kit following the manufacturer’s protocol and eluted into 10 μl EB buffer. The transcriptome sequencing (RNA-seq) library was then processed using a NEBNext ultradirectional RNA library prep kit for Illumina (NEB, USA) following the manufacturer’s recommendations. A 5-μl volume of depleted RNA mix was used together with 5 μl of first-strand synthesis reaction mix (NEBNext first-strand synthesis reaction buffer [5×] and NEBNext random primers), fragmented by heating at 94°C for the desired time. This step is followed by first-strand cDNA synthesis performed using reverse transcriptase and oligo(dT) primers. Synthesis of double-stranded (ds) cDNA is performed using the second-strand master mix provided in the kit, followed by end repair and adaptor ligation. Finally, the library is enriched (each library has a unique barcode, and each primer has a common adaptor sequence which was added in the previous adaptor ligation step and a unique overhang index unique to each sample) by a certain number of cycles of amplification and purified by the use of Agencourt AMPure beads (Beckman Coulter, catalog no. A63881). Finally, individual libraries were pooled with equimolar volumes and sequenced by the use of an Illumina HiSeq 3000 system (Illumina Inc., CA, US) and a run of 2 × 100 cycles.

### Illumina instrument run.

In preparation for sequencing, barcoded libraries were sized on an Agilent 2200 TapeStation system. Quantitation was done by the use of both QUBIT and qPCR (Kapa Biosystems, catalog no. KK4824). Individual samples were pooled in equimolar volumes at 2.5 nM. This “working pool” was used as the input in the HiSeq 3000 instrument sample preparation protocol (Illumina material no. 20015630, document no. 15066496 v04, January 2017). Typically, a 250 pM library concentration was used for clustering on a cBOT amplification system. This resulted in an optimum clustering density at which the proportions of clusters passing the filters ranged between 65% and 75%. Six RNA-seq barcoded libraries were pooled for sequencing in multiplex on a single flow cell lane, using a configuration of 2 × 100 (paired-end) cycles. Such an sequencing configuration was achieved by pooling the reagents from 150-cycle and 50-cycle Illumina HiSeq 3000 SBS kits. A typical sequencing run in the HiSeq 3000 instrument produced >300 million reads from each end, per lane, with a Q30 value of ≥90%. For RNA-seq, the use of 50 million reads per end per sample provided sufficient depth for transcriptome analysis. The sequencing run was performed at the NextGen research facility of the Interdisciplinary Center for Biotechnology Research (University of Florida).

### Assessment of cytokine and chemokine production.

Frozen cell culture supernatant fluids were thawed, and the levels of TNF-α, IL-1β, IL-6, IL-8, IL-10, and RANTES were determined by the use of Milliplex multiplex assays (EMD, Millipore). Data were acquired on a Luminex 200 system running xPONENT 3.1 software (Luminex) and analyzed using a 5-parameter logistic spline-curve fitting method and Milliplex Analyst V5.1 software (Vigene Tech). Statistical differences were assessed by two-way analysis of variance (ANOVA) using the ‘emmeans’ package in R, applying an FDR value of <0.05 for multiple-comparison corrections. Experiments were performed in triplicate.

### Galleria mellonella infection model.

For all of the G. mellonella experiments, insects in the final instar larval stage were purchased from Vanderhorst, Inc. (St. Marys, OH). Upon arrival, any dead larvae present were separated from healthy larvae, which were then weighed and placed randomly into groups and kept at room temperature until the next day, when infection was performed. Seven groups of 15 larvae, ranging in weight from 200 to 300 mg, and with no signs of melanization, were randomly chosen and used for subsequent infection. A 25-μl Hamilton syringe was used to inject 5-μl aliquots of bacterial inoculum into each larva's hemocoel via the last left proleg. Three groups received wild-type P. gingivalis (3.85 × 10^8^, 7.7 × 10^7^, and 3.85 × 10^6^ CFU per larvae), and three groups received the *cas*3 mutant (2.15 × 10^8^, 4.3 × 10^7^, and 2.15 × 10^6^ CFU per larvae). Three control groups included THSB medium alone, tryptic soy broth (TSB) (BD, Becton, Dickinson and Co.) plus P. gingivalis wild-type heat-killed (10 min at 75°C), and THSB plus Δ*cas*3 mutant heat-killed (10 min at 75°C). After injection, larvae were incubated at 37°C, and the appearance of melanization and survival were recorded at 0.5, 1, 1.5, 2, 3, 3.75, 4.25, 6.25, 22, 24, 28, and 42 h. After injection, larvae were incubated in the dark at 37°C, and appearance (signs of melanization) and survival were recorded at selected intervals. Larvae were scored as dead when they displayed no movement in response to touch. Kaplan-Meier killing curves were plotted, and estimations of differences in survival were compared using a log-rank test. A *P* value of ≤0.05 was considered significant. All data were analyzed with the ‘survival’ and ‘survminer’ packages in R. Experiments were repeated three independent times with similar results.

We followed the protocol described above for the infection transcriptome experiments, but the initial concentrations of P. gingivalis injected were 7.0 × 10^8^ CFU/ml for the wild-type strain and 1.0 × 10^8^ CFU/ml for the mutant strain.

### Host-bacterium transcriptome analysis.

We used the PATRIC annotation for genome identifier (ID) 431947.7 of P. gingivalis 33277 (82) for our study. Low-quality sequences were removed from the query files using Trimmomatic (85). Cleaned data were aligned against the P. gingivalis ATCC 33277 genome database using bowtie2 (86). Eukaryotic sequences, human and G. mellonella, were aligned against genome release 33 (GRCh38.p13) in GenCode (https://www.gencodegenes.org/) and RefSeq assembly accession no. GCF_003640425.2, respectively. Alignment for eukaryotic sequences was performed using STAR (87). Read counts from the BAM files were obtained using featureCounts (88).

In the case of the THP-1 infection experiments, differential expression analysis was performed using NOISeqBio (89). After exploratory analysis with NOISeqBio, we selected reads per kilobase per million (RPKM) normalization for the THP-1 cell data, tmm normalization with length correction for the P. gingivalis intracellular transcriptome, and tmm normalization without length correction for the P. gingivalis planktonic transcriptome. Posterior analyses were used only with significant differential expression and log changes >2.

The genome of G. mellonella has been sequenced and contains 14,067 protein-coding genes (90). Time-series analysis of the G. mellonella infection transcriptomes was performed in two steps. First, we identified differentially expressed genes using a DESeq2 spline approach, as recommended by Spies et al. for short-time series data (<8 time points) (91). In the second step, we clustered those genes based on their trajectories during the experimental period. For the following step, we used the Dirichlet process Gaussian process mixture model (DPGP) and DP_GP cluster software with 500 iterations (92).

We used Gene Ontology (GO) terms for gene set enrichment analysis (GSEA). In the case of P. gingivalis and G. mellonella, we generated our own GO annotation using the pipeline in OmicsBox (93). The GO annotation was extracted for the human genome directly from its gff3 file. GSEA was performed using GOrilla (94) in the case of the human sequences and OmicsBox for any other enrichment analysis, including enrichment using Fisher’s exact test. In all cases, we consider an FDR value of <0.05. Summaries of GO terms and GO treemaps were obtained using REVIGO (95). GSEA results corresponding to enriched terms from OmixBox, quantified as percentages of sequences in the reference and test sets, were represented as heat maps using the ‘pheatmap’ package in R, after normalization with the function ‘decostand’ from the ‘vegan’ package.

### Coexpression networks of host and P. gingivalis genes.

The integration of host-microbe expression profiles was performed using the R package ‘mixOmics’ (96). We calculated the sparse partial least-square (sPLS) correlations between the differentially expressed genes from eukaryotic cells and the different mutants of P. gingivalis. Relevance networks showing correlations between genes from eukaryotic cells and microorganisms were visualized in Cytoscape (97) with a threshold correlation of 0.90.

We used the Cytoscape plugin Diffany (41) to obtain and analyze consensus and differential coexpression networks. On those networks, we visualized enriched GO terms on the G. mellonella genes at the biological process level using the Cytoscape plugin GOlorize (98), which uses the Cytoscape BiNGO (99) plugin to perform GSEA on the nodes of the network. For GSEA performed on BiNGO, we generated the BiNGO annotation based on the GO annotation described above. The tests were performed using the default settings with an FDR significance value of <0.05. The selection of the enriched terms to be visualized was performed by summarizing the results with REVIGO and selecting for the representative GO terms. In the case of P. gingivalis genes present in the networks, we associated them with GO terms, removed the singletons, and summarized the results using REVIGO.

### Data availability.

The data sets used in these analyses were deposited at the Gene Expression Omnibus (GEO) data repository of NCBI. Sequences have been deposited at the GEO database (https://www.ncbi.nlm.nih.gov/geo/) with submission number GSE154569.

## References

[1] Makarova KS, Wolf YI, Iranzo J, Shmakov SA, Alkhnbashi OS, Brouns SJJ, Charpentier E, Cheng D, Haft DH, Horvath P, Moineau S, Mojica FJM, Scott D, Shah SA, Siksnys V, Terns MP, Venclovas Č, White MF, Yakunin AF, Yan W, Zhang F, Garrett RA, Backofen R, van der Oost J, Barrangou R, Koonin EV. 2020. Evolutionary classification of CRISPR-Cas systems: a burst of class 2 and derived variants. Nat Rev Microbiol 18:67–83. doi:10.1038/s41579-019-0299-x. 31857715 PMC8905525

[2] Hampton HG, Watson BNJ, Fineran PC. 2020. The arms race between bacteria and their phage foes. Nature 577:327–336. doi:10.1038/s41586-019-1894-8. 31942051

[3] Jackson SA, McKenzie RE, Fagerlund RD, Kieper SN, Fineran PC, Brouns SJJ. 2017. CRISPR-Cas: adapting to change. Science 356:eaal5056. doi:10.1126/science.aal5056. 28385959

[4] Makarova KS, Anantharaman V, Aravind L, Koonin EV. 2012. Live virus-free or die: coupling of antivirus immunity and programmed suicide or dormancy in prokaryotes. Biol Direct 7:40. doi:10.1186/1745-6150-7-40. 23151069 PMC3506569

[5] Perez-Rodriguez R, Haitjema C, Huang Q, Nam KH, Bernardis S, Ke A, DeLisa MP. 2011. Envelope stress is a trigger of CRISPR RNA-mediated DNA silencing in Escherichia coli. Mol Microbiol 79:584–599. doi:10.1111/j.1365-2958.2010.07482.x. 21255106 PMC3040579

[6] Louwen R, Horst-Kreft D, Boer AG, Graaf L, Knegt G, Hamersma M, Heikema AP, Timms AR, Jacobs BC, Wagenaar JA, Endtz HP, Oost J, Wells JM, Nieuwenhuis EES, Vliet AHM, Willemsen PTJ, Baarlen P, Belkum A. 2013. A novel link between Campylobacter jejuni bacteriophage defence, virulence and Guillain-Barré syndrome. Eur J Clin Microbiol Infect Dis 32:207–226. doi:10.1007/s10096-012-1733-4. 22945471

[7] Sampson TR, Saroj SD, Llewellyn AC, Tzeng Y-L, Weiss DS. 2013. A CRISPR/Cas system mediates bacterial innate immune evasion and virulence. Nature 497:254–257. doi:10.1038/nature12048. 23584588 PMC3651764

[8] Li R, Fang L, Tan S, Yu M, Li X, He S, Wei Y, Li G, Jiang J, Wu M. 2016. Type I CRISPR-Cas targets endogenous genes and regulates virulence to evade mammalian host immunity. Cell Res 26:1273–1287. doi:10.1038/cr.2016.135. 27857054 PMC5143421

[9] Westra ER, Buckling A, Fineran PC. 2014. CRISPR-Cas systems: beyond adaptive immunity. Nat Rev Microbiol 12:317–326. doi:10.1038/nrmicro3241. 24704746

[10] Grenier D, La VD. 2011. Proteases of Porphyromonas gingivalis as important virulence factors in periodontal disease and potential targets for plant-derived compounds: a review article. Curr Drug Targets 12:322–331. doi:10.2174/138945011794815310. 20955149

[11] Holt SC, Kesavalu L, Walker S, Genco CA. 1999. Virulence factors of Porphyromonas gingivalis. Periodontol 2000 20:168–238. doi:10.1111/j.1600-0757.1999.tb00162.x. 10522227

[12] Bender P, Bürgin WB, Sculean A, Eick S. 2017. Serum antibody levels against Porphyromonas gingivalis in patients with and without rheumatoid arthritis - a systematic review and meta-analysis. Clin Oral Invest 21:33–42. doi:10.1007/s00784-016-1938-5. 27561661

[13] Gibson FC, Genco CA. 2007. Porphyromonas gingivalis mediated periodontal disease and atherosclerosis: disparate diseases with commonalities in pathogenesis through TLRs. Curr Pharm Des 13:3665–3675. doi:10.2174/138161207783018554. 18220804

[14] Chen T, Olsen I. 2019. Porphyromonas gingivalis and its CRISPR-Cas system. J Oral Microbiol 11:1638196. doi:10.1080/20002297.2019.1638196. 31303969 PMC6609313

[15] Chen T, Siddiqui H, Olsen I. 2015. Comparative genomics and proteomics of 13 Porphyromonas gingivalis strains. J Oral Microbiol 7:29008. doi:10.3402/jom.v7.29008. 26387643 PMC4576511

[16] Chen T, Siddiqui H, Olsen I. 2017. In silico comparison of 19 Porphyromonas gingivalis strains in genomics, phylogenetics, phylogenomics and functional genomics. Front Cell Infect Microbiol 7:28. doi:10.3389/fcimb.2017.00028. 28261563 PMC5306136

[17] Burmistrz M, Dudek B, Staniec D, Rodriguez Martinez JI, Bochtler M, Potempa J, Pyrc K. 2015. Functional analysis of Porphyromonas gingivalis W83 CRISPR-Cas systems. J Bacteriol 197:2631–2641. doi:10.1128/JB.00261-15. 26013482 PMC4507336

[18] Phillips P, Progulske-Fox A, Grieshaber S, Grieshaber N. 2014. Expression of Porphyromonas gingivalis small RNA in response to hemin availability identified using microarray and RNA-seq analysis. FEMS Microbiol Lett 351:202–208. doi:10.1111/1574-6968.12320. 24245974 PMC4009720

[19] McLean JS, Lombardo M-J, Ziegler MG, Novotny M, Yee-Greenbaum J, Badger JH, Tesler G, Nurk S, Lesin V, Brami D, Hall AP, Edlund A, Allen LZ, Durkin S, Reed S, Torriani F, Nealson KH, Pevzner PA, Friedman R, Venter JC, Lasken RS. 2013. Genome of the pathogen Porphyromonas gingivalis recovered from a biofilm in a hospital sink using a high-throughput single-cell genomics platform. Genome Res 23:867–877. doi:10.1101/gr.150433.112. 23564253 PMC3638142

[20] Yost S, Duran-Pinedo AE, Teles R, Krishnan K, Frias-Lopez J. 2015. Functional signatures of oral dysbiosis during periodontitis progression revealed by microbial metatranscriptome analysis. Genome Med 7:27. doi:10.1186/s13073-015-0153-3. 25918553 PMC4410737

[21] Socransky SS, Haffajee AD, Cugini MA, Smith C, Kent RLJ. 1998. Microbial complexes in subgingival plaque. J Clin Periodontol 25:134–144. doi:10.1111/j.1600-051x.1998.tb02419.x. 9495612

[22] Rath D, Amlinger L, Rath A, Lundgren M. 2015. The CRISPR-Cas immune system: biology, mechanisms and applications. Biochimie 117:119–128. doi:10.1016/j.biochi.2015.03.025. 25868999

[23] Sztukowska M, Bugno M, Potempa J, Travis J, Kurtz DM, Jr. 2002. Role of rubrerythrin in the oxidative stress response of Porphyromonas gingivalis. Mol Microbiol 44:479–488. doi:10.1046/j.1365-2958.2002.02892.x. 11972784

[24] Diaz PI, Slakeski N, Reynolds EC, Morona R, Rogers AH, Kolenbrander PE. 2006. Role of oxyR in the oral anaerobe Porphyromonas gingivalis. J Bacteriol 188:2454–2462. doi:10.1128/JB.188.7.2454-2462.2006. 16547032 PMC1428421

[25] Lewis JP. 2010. Metal uptake in host-pathogen interactions: role of iron in Porphyromonas gingivalis interactions with host organisms. Periodontol 2000 52:94–116. doi:10.1111/j.1600-0757.2009.00329.x. 20017798 PMC2825758

[26] Ueshima J, Shoji M, Ratnayake DB, Abe K, Yoshida S, Yamamoto K, Nakayama K. 2003. Purification, gene cloning, gene expression, and mutants of Dps from the obligate anaerobe Porphyromonas gingivalis. Infect Immun 71:1170–1178. doi:10.1128/iai.71.3.1170-1178.2003. 12595429 PMC148816

[27] Aruni AW, Robles A, Fletcher HM. 2013. VimA mediates multiple functions that control virulence in Porphyromonas gingivalis. Mol Oral Microbiol 28:167–180. doi:10.1111/omi.12017. 23279905 PMC3625487

[28] Chen W, Honma K, Sharma A, Kuramitsu HK. 2006. A universal stress protein of Porphyromonas gingivalis is involved in stress responses and biofilm formation. FEMS Microbiol Lett 264:15–21. doi:10.1111/j.1574-6968.2006.00426.x. 17020544

[29] Olczak T, Simpson W, Liu X, Genco CA. 2005. Iron and heme utilization in Porphyromonas gingivalis. FEMS Microbiol Rev 29:119–144. doi:10.1016/j.femsre.2004.09.001. 15652979

[30] Kikuchi Y, Ohara N, Sato K, Yoshimura M, Yukitake H, Sakai E, Shoji M, Naito M, Nakayama K. 2005. Novel stationary-phase-upregulated protein of Porphyromonas gingivalis influences production of superoxide dismutase, thiol peroxidase and thioredoxin. Microbiology (Reading) 151:841–853. doi:10.1099/mic.0.27589-0. 15758230

[31] Haruyama K, Yoshimura A, Naito M, Kishimoto M, Shoji M, Abiko Y, Hara Y, Nakayama K. 2009. Identification of a gingipain-sensitive surface ligand of Porphyromonas gingivalis that induces Toll-like receptor 2- and 4-independent NF-κB activation in CHO cells. Infect Immun 77:4414–4420. doi:10.1128/IAI.00140-09. 19667049 PMC2747925

[32] Luan X, Zhou X, Naqvi A, Francis M, Foyle D, Nares S, Diekwisch TGH. 2018. MicroRNAs and immunity in periodontal health and disease. Int J Oral Sci 10:24. doi:10.1038/s41368-018-0025-y. 30078842 PMC6080405

[33] Xie Y, Shu R, Jiang S, Liu D, Zhang X. 2011. Comparison of microRNA profiles of human periodontal diseased and healthy gingival tissues. Int J Oral Sci 3:125–134. doi:10.4248/IJOS11046. 21789961 PMC3470093

[34] Brennan M, Thomas DY, Whiteway M, Kavanagh K. 2002. Correlation between virulence of Candida albicans mutants in mice and Galleria mellonella larvae. FEMS Immunol Med Microbiol 34:153–157. doi:10.1111/j.1574-695X.2002.tb00617.x. 12381467

[35] Jander G, Rahme LG, Ausubel FM. 2000. Positive correlation between virulence of Pseudomonas aeruginosa mutants in mice and insects. J Bacteriol 182:3843–3845. doi:10.1128/jb.182.13.3843-3845.2000. 10851003 PMC94559

[36] Arteaga Blanco LA, Crispim JS, Fernandes KM, de Oliveira LL, Pereira MF, Bazzolli DMS, Martins GF. 2017. Differential cellular immune response of Galleria mellonella to Actinobacillus pleuropneumoniae. 1. Cell Tissue Res 370:153–168. doi:10.1007/s00441-017-2653-5. 28687931

[37] Giannouli M, Palatucci AT, Rubino V, Ruggiero G, Romano M, Triassi M, Ricci V, Zarrilli R. 2014. Use of larvae of the wax moth Galleria mellonella as an in vivo model to study the virulence of Helicobacter pylori. BMC Microbiol 14:228. doi:10.1186/s12866-014-0228-0. 25170542 PMC4148543

[38] dos Santos JD, de Alvarenga JA, Rossoni RD, García MT, Moraes RM, Anbinder AL, Cardoso Jorge AO, Junqueira JC. 2017. Immunomodulatory effect of photodynamic therapy in Galleria mellonella infected with Porphyromonas gingivalis. Microb Pathog 110:507–511. doi:10.1016/j.micpath.2017.07.045. 28757273

[39] Clark KD. 2020. Insect hemolymph immune complexes, p 123–161. *In* Hoeger U, Harris JR (ed), Vertebrate and invertebrate respiratory proteins, lipoproteins and other body fluid proteins. Springer International Publishing, Cham, Switzerland.

[40] Söderhäll K, Cerenius L. 1998. Role of the prophenoloxidase-activating system in invertebrate immunity. Curr Opin Immunol 10:23–28. doi:10.1016/s0952-7915(98)80026-5. 9523106

[41] Van Landeghem S, Van Parys T, Dubois M, Inzé D, Van de Peer Y. 2016. Diffany: an ontology-driven framework to infer, visualise and analyse differential molecular networks. BMC Bioinformatics 17:18. doi:10.1186/s12859-015-0863-y. 26729218 PMC4700732

[42] Lum AG, Ly M, Santiago-Rodriguez TM, Naidu M, Boehm TK, Pride DT. 2015. Global transcription of CRISPR loci in the human oral cavity. BMC Genomics 16:401. doi:10.1186/s12864-015-1615-0. 25994215 PMC4438527

[43] Gong T, Zeng J, Tang B, Zhou X, Li Y. 2020. CRISPR-Cas systems in oral microbiome: from immune defense to physiological regulation. Mol Oral Microbiol 35:41–48. doi:10.1111/omi.12279. 31995666

[44] Hitch G, Pratten J, Taylor PW. 2004. Isolation of bacteriophages from the oral cavity. Lett Appl Microbiol 39:215–219. doi:10.1111/j.1472-765X.2004.01565.x. 15242464

[45] Szafrański SP, Winkel A, Stiesch M. 2017. The use of bacteriophages to biocontrol oral biofilms. J Biotechnol 250:29–44. doi:10.1016/j.jbiotec.2017.01.002. 28108235

[46] Gunderson FF, Mallama CA, Fairbairn SG, Cianciotto NP. 2015. Nuclease activity of Legionella pneumophila Cas2 promotes intracellular infection of amoebal host cells. Infect Immun 83:1008–1018. doi:10.1128/IAI.03102-14. 25547789 PMC4333442

[47] Sampson TR, Weiss DS. 2014. CRISPR-Cas systems: new players in gene regulation and bacterial physiology. Front Cell Infect Microbiol 4:37. doi:10.3389/fcimb.2014.00037. 24772391 PMC3983513

[48] Li L, Michel R, Cohen J, DeCarlo A, Kozarov E. 2008. Intracellular survival and vascular cell-to-cell transmission of Porphyromonas gingivalis. BMC Microbiol 8:26. doi:10.1186/1471-2180-8-26. 18254977 PMC2259307

[49] Rudney JD, Chen R, Sedgewick GJ. 2005. Actinobacillus actinomycetemcomitans, Porphyromonas gingivalis, and Tannerella forsythensis are components of a polymicrobial intracellular flora within human buccal cells. J Dent Res 84:59–63. doi:10.1177/154405910508400110. 15615877

[50] Noiri Y, Ozaki K, Nakae H, Matsuo T, Ebisu S. 1997. An immunohistochemical study on the localization of Porphyromonas gingivalis, Campylobacter rectus and Actinomyces viscosus in human periodontal pockets. J Periodontal Res 32:598–607. doi:10.1111/j.1600-0765.1997.tb00937.x. 9401932

[51] Yilmaz Ö, Verbeke P, Lamont RJ, Ojcius DM. 2006. Intercellular spreading of Porphyromonas gingivalis infection in primary gingival epithelial cells. Infect Immun 74:703–710. doi:10.1128/IAI.74.1.703-710.2006. 16369027 PMC1346639

[52] Miller DP, Hutcherson JA, Wang Y, Nowakowska ZM, Potempa J, Yoder-Himes DR, Scott DA, Whiteley M, Lamont RJ. 2017. Genes contributing to Porphyromonas gingivalis fitness in abscess and epithelial cell colonization environments. Front Cell Infect Microbiol 7:378. doi:10.3389/fcimb.2017.00378. 28900609 PMC5581868

[53] Cui L, Wang X, Huang D, Zhao Y, Feng J, Lu Q, Pu Q, Wang Y, Cheng G, Wu M, Dai M. 2020. CRISPR-cas3 of Salmonella upregulates bacterial biofilm formation and virulence to host cells by targeting quorum-sensing systems. Pathogens 9:53. doi:10.3390/pathogens9010053. PMC7168661 31936769

[54] Mydel P, Takahashi Y, Yumoto H, Sztukowska M, Kubica M, Gibson FC, Kurtz DM, Travis J, Collins LV, Nguyen K-A, Genco CA, Potempa J. 2006. Roles of the host oxidative immune response and bacterial antioxidant rubrerythrin during Porphyromonas gingivalis infection. PLoS Pathog 2:e76. doi:10.1371/journal.ppat.0020076. 16895445 PMC1522038

[55] Seers CA, Slakeski N, Veith PD, Nikolof T, Chen Y-Y, Dashper SG, Reynolds EC. 2006. The RgpB C-terminal domain has a role in attachment of RgpB to the outer membrane and belongs to a novel C-terminal-domain family found in Porphyromonas gingivalis. J Bacteriol 188:6376–6386. doi:10.1128/JB.00731-06. 16923905 PMC1595369

[56] Watanabe T, Nozawa T, Aikawa C, Amano A, Maruyama F, Nakagawa I. 2013. CRISPR regulation of intraspecies diversification by limiting IS transposition and intercellular recombination. Genome Biol Evol 5:1099–1114. doi:10.1093/gbe/evt075. 23661565 PMC3698921

[57] Lionakis MS. 2011. Drosophila and Galleria insect model hosts. Virulence 2:521–527. doi:10.4161/viru.2.6.18520. 22186764 PMC3260546

[58] Mukherjee K, Altincicek B, Hain T, Domann E, Vilcinskas A, Chakraborty T. 2010. Galleria mellonella as a model system for studying listeria pathogenesis. Appl Environ Microbiol 76:310–317. doi:10.1128/AEM.01301-09. 19897755 PMC2798647

[59] Lange A, Schäfer A, Frick J-S. 2019. A Galleria mellonella oral administration model to study commensal-induced innate immune responses. J Vis Exp doi:10.3791/59270. 30958466

[60] Rossoni RD, de Ribeiro FC, Dos Santos HFS, Dos Santos JD, de Oliveira NS, Dutra MTDS, de Lapena SAB, Junqueira JC. 2019. Galleria mellonella as an experimental model to study human oral pathogens. Arch Oral Biol 101:13–22. doi:10.1016/j.archoralbio.2019.03.002. 30856377

[61] Stobernack T, Du Teil Espina M, Mulder LM, Palma Medina LM, Piebenga DR, Gabarrini G, Zhao X, Janssen KMJ, Hulzebos J, Brouwer E, Sura T, Becher D, van Winkelhoff AJ, Götz F, Otto A, Westra J, van Dijl JM. 2018. A secreted bacterial peptidylarginine deiminase can neutralize human innate immune defenses. mBio 9:e01704-18. doi:10.1128/mBio.01704-18. 30377277 PMC6212822

[62] Bélanger M, Rodrigues PH, Dunn William A, Jr, Progulske-Fox A. 2006. Autophagy: a highway for Porphyromonas gingivalis in endothelial cells. Autophagy 2:165–170. doi:10.4161/auto.2828. 16874051

[63] Rodrigues PH, Bélanger M, Dunn W, Progulske-Fox A. 2008. Porphyromonas gingivalis and the autophagic pathway: an innate immune interaction? Front Biosci 13:178–187. doi:10.2741/2668. 17981536

[64] Hirasawa M, Kurita-Ochiai T. 2018. Porphyromonas gingivalis induces apoptosis and autophagy via ER stress in human umbilical vein endothelial cells. Mediators Inflamm 2018:1967506. doi:10.1155/2018/1967506. 30150893 PMC6087591

[65] Cutuli MA, Petronio Petronio G, Vergalito F, Magnifico I, Pietrangelo L, Venditti N, Di Marco R. 2019. Galleria mellonella as a consolidated in vivo model hosts: new developments in antibacterial strategies and novel drug testing. Virulence 10:527–541. doi:10.1080/21505594.2019.1621649. 31142220 PMC6550544

[66] Pereira TC, de Barros PP, de Fugisaki LRO, Rossoni RD, de Ribeiro FC, de Menezes RT, Junqueira JC, Scorzoni L. 2018. Recent advances in the use of Galleria mellonella model to study immune responses against human pathogens. J Fungi 4:128. doi:10.3390/jof4040128. PMC6308929 30486393

[67] Wojda I. 2017. Immunity of the greater wax moth Galleria mellonella. Insect Sci 24:342–357. doi:10.1111/1744-7917.12325. 26847724

[68] Gorr S-U, Abdolhosseini M. 2011. Antimicrobial peptides and periodontal disease. J Clin Periodontol 38(Suppl 11):126–141. doi:10.1111/j.1600-051X.2010.01664.x. 21323710

[69] Gorr S-U. 2012. Antimicrobial peptides in periodontal innate defense. Front Oral Biol 15:84–98. doi:10.1159/000329673. 22142958 PMC3704226

[70] Correa W, Manrique-Moreno M, Behrends J, Patiño E, Marella C, Peláez-Jaramillo C, Garidel P, Gutsmann T, Brandenburg K, Heinbockel L. 2014. Galleria mellonella native and analogue peptides Gm1 and ΔGm1. II) anti-bacterial and anti-endotoxic effects. Biochim Biophys Acta 1838:2739–2744. doi:10.1016/j.bbamem.2014.07.005. 25016054

[71] Tsai CJ-Y, Loh JMS, Proft T. 2016. Galleria mellonella infection models for the study of bacterial diseases and for antimicrobial drug testing. Virulence 7:214–229. doi:10.1080/21505594.2015.1135289. 26730990 PMC4871635

[72] Zdybicka-Barabas A, Stączek S, Pawlikowska-Pawlęga B, Mak P, Luchowski R, Skrzypiec K, Mendyk E, Wydrych J, Gruszecki WI, Cytryńska M. 2019. Studies on the interactions of neutral Galleria mellonella cecropin D with living bacterial cells. Amino Acids 51:175–191. doi:10.1007/s00726-018-2641-4. 30167962

[73] Chertkova EA, Grizanova EV, Dubovskiy IM. 2018. Bacterial and fungal infections induce bursts of dopamine in the haemolymph of the Colorado potato beetle Leptinotarsa decemlineata and greater wax moth Galleria mellonella. J Invertebr Pathol 153:203–206. doi:10.1016/j.jip.2018.02.020. 29501498

[74] Wittwer D, Franchini A, Ottaviani E, Wiesner A. 1999. Presence of IL-1- and TNF-like molecules in Galleria mellonella (Lepidoptera) haemocytes and in an insect cell line Fromestigmene acraea (Lepidoptera). Cytokine 11:637–642. doi:10.1006/cyto.1998.0481. 10479399

[75] Vogel H, Altincicek B, Glöckner G, Vilcinskas A. 2011. A comprehensive transcriptome and immune-gene repertoire of the lepidopteran model host Galleria mellonella. BMC Genomics 12:308. doi:10.1186/1471-2164-12-308. 21663692 PMC3224240

[76] Weber ANR, Tauszig-Delamasure S, Hoffmann JA, Lelièvre E, Gascan H, Ray KP, Morse MA, Imler J-L, Gay NJ. 2003. Binding of the Drosophila cytokine Spätzle to Toll is direct and establishes signaling. Nat Immunol 4:794–800. doi:10.1038/ni955. 12872120

[77] Mizerska‐Dudka M, Andrejko M. 2014. Galleria mellonella hemocytes destruction after infection with Pseudomonas aeruginosa. 3. J Basic Microbiol 54:232–246. doi:10.1002/jobm.201200273. 23456635

[78] Jostins L, Ripke S, Weersma RK, Duerr RH, McGovern DP, Hui KY, Lee JC, Schumm LP, Sharma Y, Anderson CA, Essers J, Mitrovic M, Ning K, Cleynen I, Theatre E, Spain SL, Raychaudhuri S, Goyette P, Wei Z, Abraham C, Achkar J-P, Ahmad T, Amininejad L, Ananthakrishnan AN, Andersen V, Andrews JM, Baidoo L, Balschun T, Bampton PA, Bitton A, Boucher G, Brand S, Büning C, Cohain A, Cichon S, D'Amato M, De Jong D, Devaney KL, Dubinsky M, Edwards C, Ellinghaus D, Ferguson LR, Franchimont D, Fransen K, Gearry R, Georges M, Gieger C, Glas J, Haritunians T, Hart A, International IBD Genetics Consortium (IIBDGC), . 2012. Host-microbe interactions have shaped the genetic architecture of inflammatory bowel disease. Nature 491:119–124. doi:10.1038/nature11582. 23128233 PMC3491803

[79] Luo G, Sun Y, Huang L, Su Y, Zhao L, Qin Y, Xu X, Yan Q. 2020. Time-resolved dual RNA-seq of tissue uncovers Pseudomonas plecoglossicida key virulence genes in host-pathogen interaction with Epinephelus coioides. Environ Microbiol 22:677–693. doi:10.1111/1462-2920.14884. 31797531

[80] Moghaddam MRB, Tonk M, Schreiber C, Salzig D, Czermak P, Vilcinskas A, Rahnamaeian M. 2016. The potential of the Galleria mellonella innate immune system is maximized by the co-presentation of diverse antimicrobial peptides. Biol Chem 397:939–945. doi:10.1515/hsz-2016-0157. 27105487

[81] Grenier D, Roy S, Chandad F, Plamondon P, Yoshioka M, Nakayama K, Mayrand D. 2003. Effect of inactivation of the Arg- and/or Lys-gingipain gene on selected virulence and physiological properties of Porphyromonas gingivalis. Infect Immun 71:4742–4748. doi:10.1128/iai.71.8.4742-4748.2003. 12874356 PMC166032

[82] Wattam AR, Davis JJ, Assaf R, Boisvert S, Brettin T, Bun C, Conrad N, Dietrich EM, Disz T, Gabbard JL, Gerdes S, Henry CS, Kenyon RW, Machi D, Mao C, Nordberg EK, Olsen GJ, Murphy-Olson DE, Olson R, Overbeek R, Parrello B, Pusch GD, Shukla M, Vonstein V, Warren A, Xia F, Yoo H, Stevens RL. 2017. Improvements to PATRIC, the all-bacterial bioinformatics database and analysis resource center. Nucleic Acids Res 45:D535–D542. doi:10.1093/nar/gkw1017. 27899627 PMC5210524

[83] Rocha FG, Moye ZD, Ottenberg G, Tang P, Campopiano DJ, Gibson FC, Davey ME. 2020. Porphyromonas gingivalis sphingolipid synthesis limits the host inflammatory response. J Dent Res 99:568–576. doi:10.1177/0022034520908784. 32105543 PMC7174802

[84] Livak KJ, Schmittgen TD. 2001. Analysis of relative gene expression data using real-time quantitative PCR and the 2(-Delta Delta C(T)) method. Methods 25:402–408. doi:10.1006/meth.2001.1262. 11846609

[85] Bolger AM, Lohse M, Usadel B. 2014. Trimmomatic: a flexible trimmer for Illumina sequence data. Bioinformatics 30:2114–2120. doi:10.1093/bioinformatics/btu170. 24695404 PMC4103590

[86] Langmead B, Salzberg SL. 2012. Fast gapped-read alignment with Bowtie 2. Nat Methods 9:357–359. doi:10.1038/nmeth.1923. 22388286 PMC3322381

[87] Dobin A, Gingeras TR. 2016. Optimizing RNA-Seq mapping with STAR. Methods Mol Biol 1415:245–262. doi:10.1007/978-1-4939-3572-7_13. 27115637

[88] Liao Y, Smyth GK, Shi W. 2014. featureCounts: an efficient general purpose program for assigning sequence reads to genomic features. Bioinformatics 30:923–930. doi:10.1093/bioinformatics/btt656. 24227677

[89] Tarazona S, García-Alcalde F, Dopazo J, Ferrer A, Conesa A. 2011. Differential expression in RNA-seq: a matter of depth. Genome Res 21:2213–2223. doi:10.1101/gr.124321.111. 21903743 PMC3227109

[90] Lange A, Beier S, Huson DH, Parusel R, Iglauer F, Frick J-S. 2018. Genome sequence of Galleria mellonella (Greater Wax Moth). Genome Announc 6:e01220-17. doi:10.1128/genomeA.01220-17. 29326202 PMC5764926

[91] Spies D, Renz PF, Beyer TA, Ciaudo C. 2019. Comparative analysis of differential gene expression tools for RNA sequencing time course data. Brief Bioinform 20:288–298. doi:10.1093/bib/bbx115. 29028903 PMC6357553

[92] McDowell IC, Manandhar D, Vockley CM, Schmid AK, Reddy TE, Engelhardt BE. 2018. Clustering gene expression time series data using an infinite Gaussian process mixture model. PLoS Comput Biol 14:e1005896. doi:10.1371/journal.pcbi.1005896. 29337990 PMC5786324

[93] BioBam Bioinformatics. 2019. OmicsBox - Bioinformatics made easy (Version 1.3.3). https://www.biobam.com/omicsbox/.

[94] Eden E, Navon R, Steinfeld I, Lipson D, Yakhini Z. 2009. GOrilla: a tool for discovery and visualization of enriched GO terms in ranked gene lists. BMC Bioinformatics 10:48. doi:10.1186/1471-2105-10-48. 19192299 PMC2644678

[95] Supek F, Bošnjak M, Škunca N, Šmuc T. 2011. REVIGO summarizes and visualizes long lists of gene ontology terms. PLoS One 6:e21800. doi:10.1371/journal.pone.0021800. 21789182 PMC3138752

[96] Rohart F, Gautier B, Singh A, Lê Cao K-A. 2017. mixOmics: an R package for ’omics feature selection and multiple data integration. PLoS Comput Biol 13:e1005752. doi:10.1371/journal.pcbi.1005752. 29099853 PMC5687754

[97] Su G, Morris JH, Demchak B, Bader GD. 2014. Biological network exploration with cytoscape 3. Curr Protoc Bioinformatics 47:8.13.1–8.13.24. doi:10.1002/0471250953.bi0813s47. 25199793 PMC4174321

[98] Garcia O, Saveanu C, Cline M, Fromont-Racine M, Jacquier A, Schwikowski B, Aittokallio T. 2007. GOlorize: a Cytoscape plug-in for network visualization with Gene Ontology-based layout and coloring. Bioinformatics 23:394–396. doi:10.1093/bioinformatics/btl605. 17127678

[99] Maere S, Heymans K, Kuiper M. 2005. BiNGO: a Cytoscape plugin to assess overrepresentation of gene ontology categories in biological networks. Bioinformatics 21:3448–3449. doi:10.1093/bioinformatics/bti551. 15972284

