# Supplementary material for: Virulence of the Pathogen Porphyromonas gingivalis Is Controlled by the CRISPR-Cas Protein Cas3
Source: mSystems. 2020 Sep 29;5(5):e00852-20. doi: 10.1128/mSystems.00852-20 (PMC7527141; doi:10.1128/mSystems.00852-20)
Supplement: FIG S1 [file mSystems.00852-20-sf001.pdf]

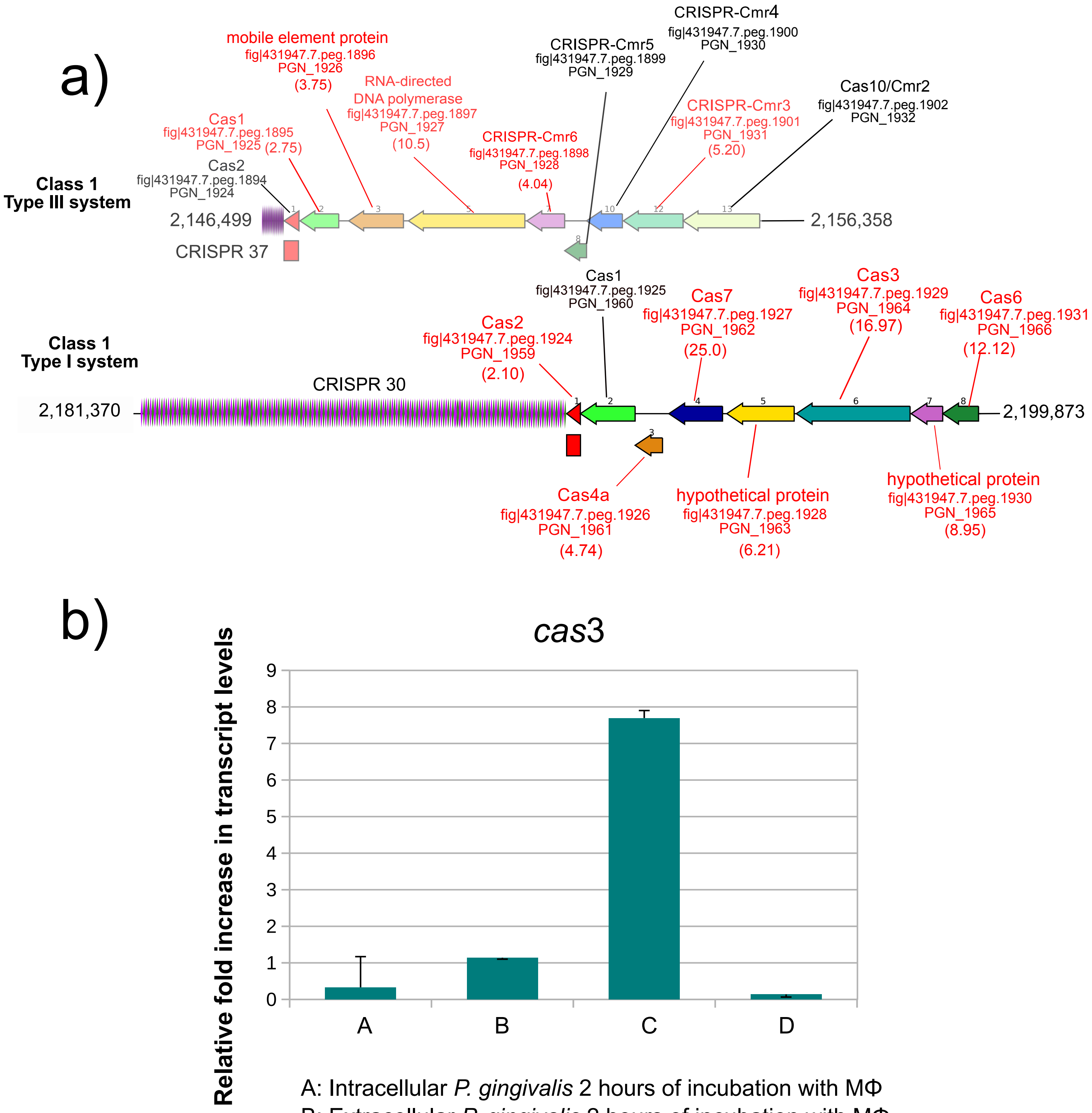

A: Intracellular *P. gingivalis* 2 hours of incubation with MΦ
B: Extracellular *P. gingivalis* 2 hours of incubation with MΦ
C: Intracellular *P. gingivalis* 6 hours of incubation with MΦ
D: Extracellular *P. gingivalis* 6 hours of incubation with MΦ
