# Supplementary figures and images for: Virulence of the Pathogen Porphyromonas gingivalis Is Controlled by the CRISPR-Cas Protein Cas3
Source: mSystems. 2020 Sep 29;5(5):e00852-20. doi: 10.1128/mSystems.00852-20 (PMC7527141; doi:10.1128/mSystems.00852-20)

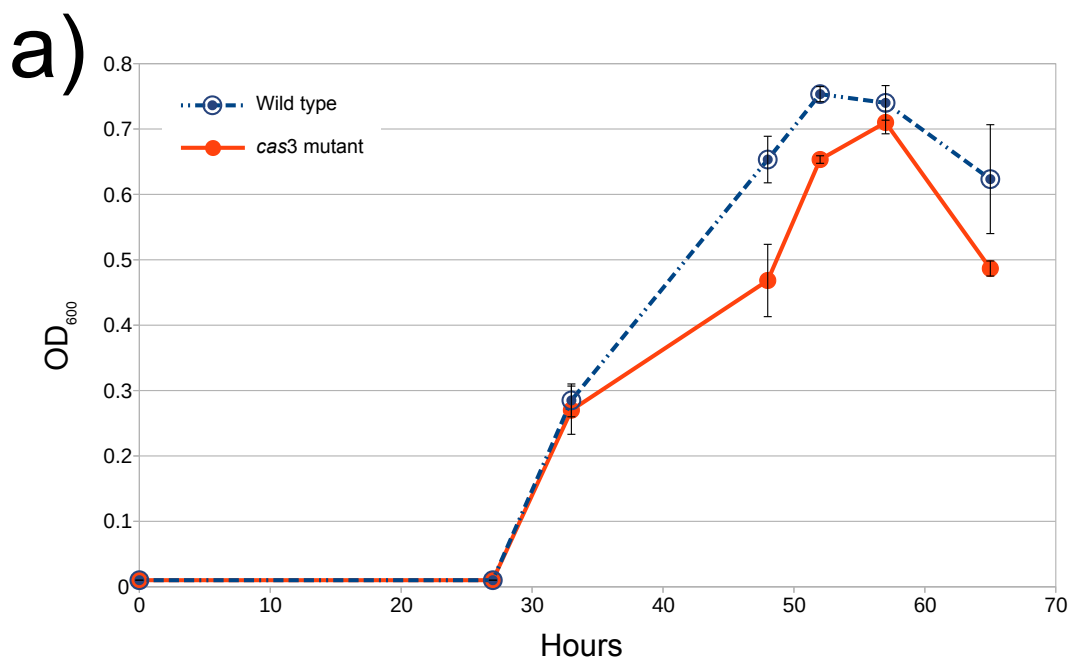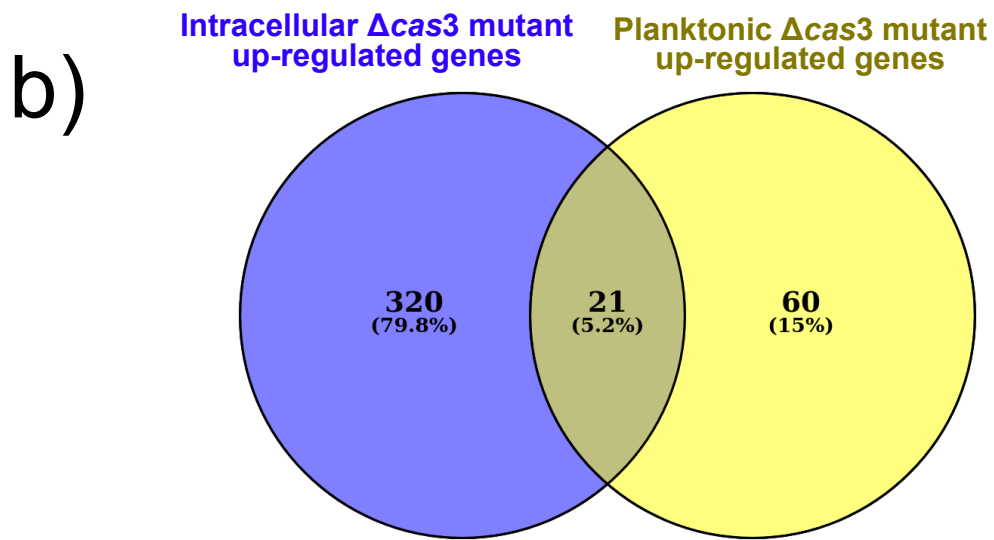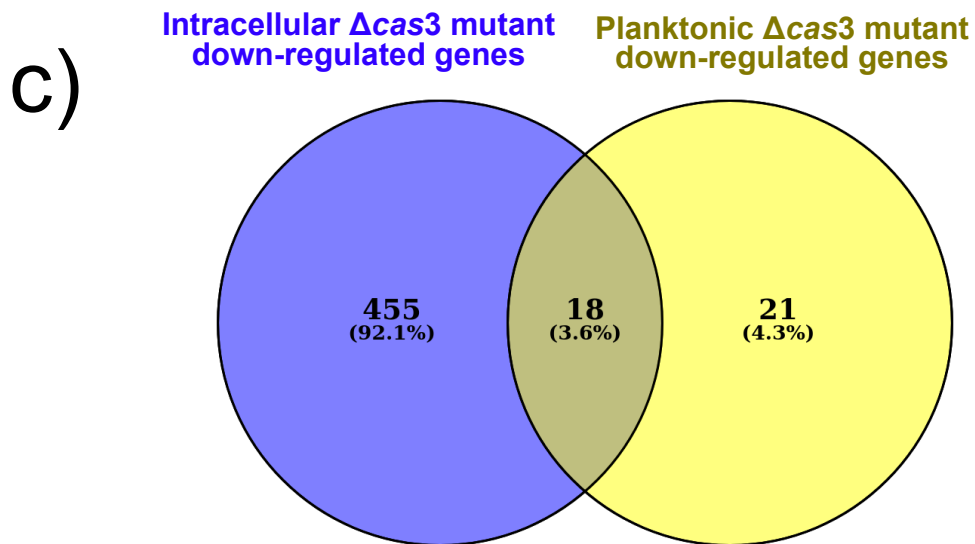

Supplement: FIG S2 [file mSystems.00852-20-sf002.pdf]

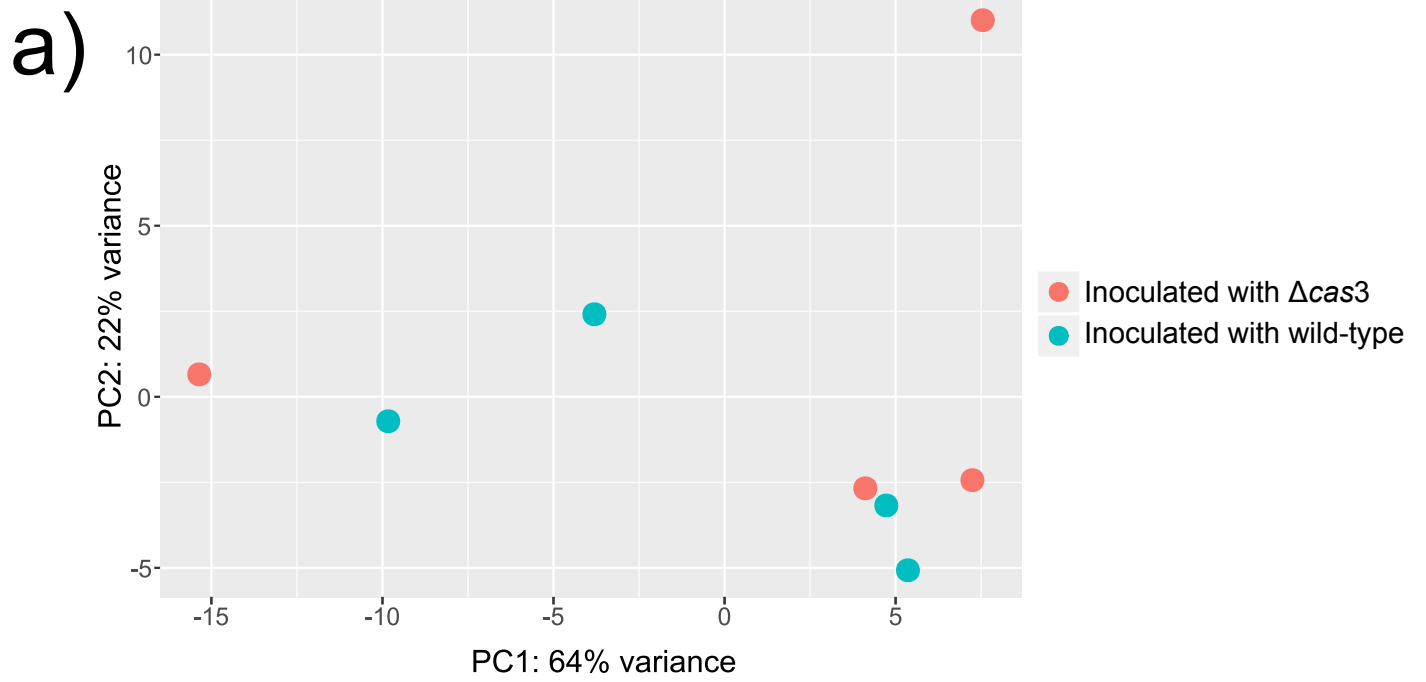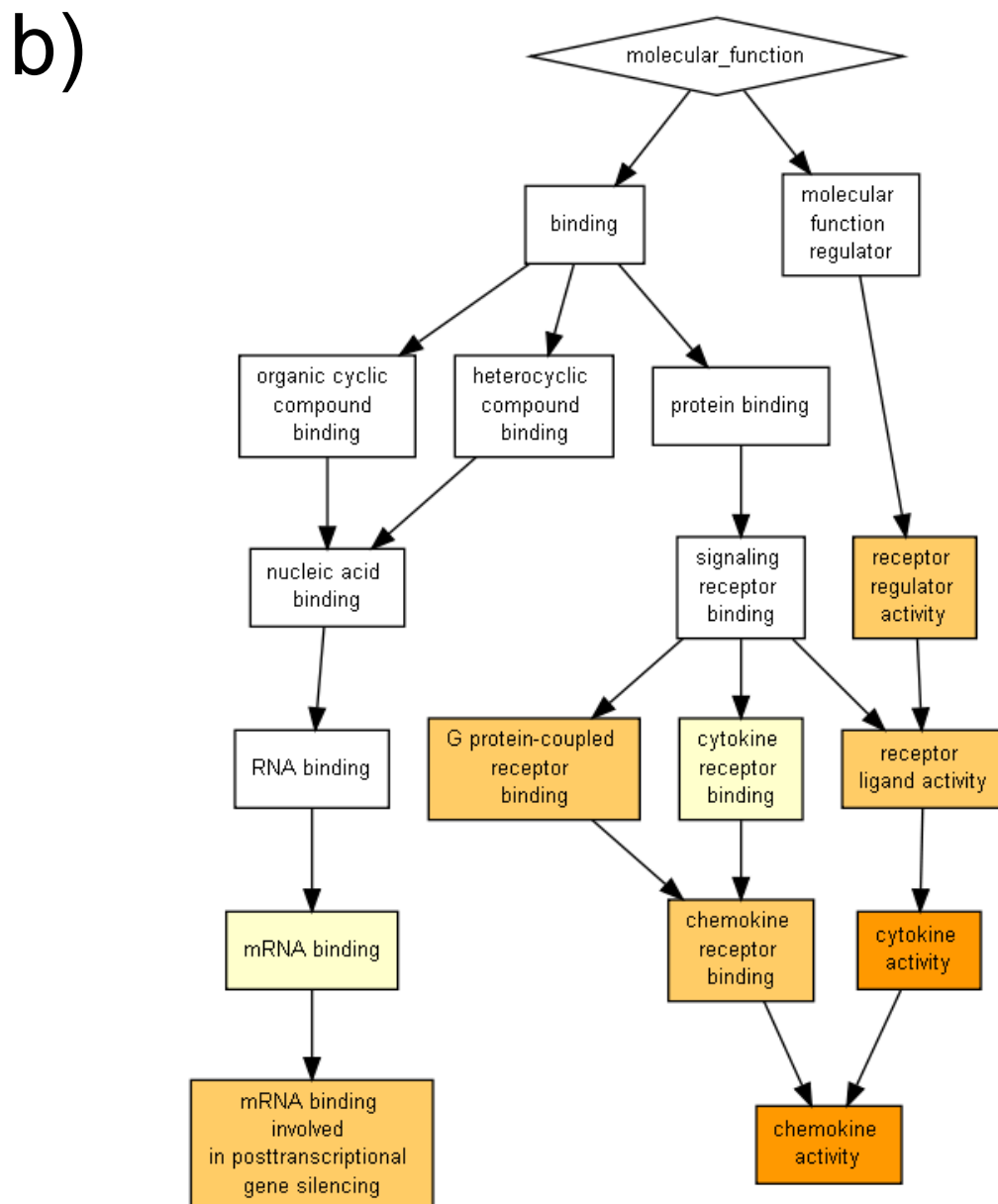

Supplement: FIG S3 [file mSystems.00852-20-sf003.pdf]

a)

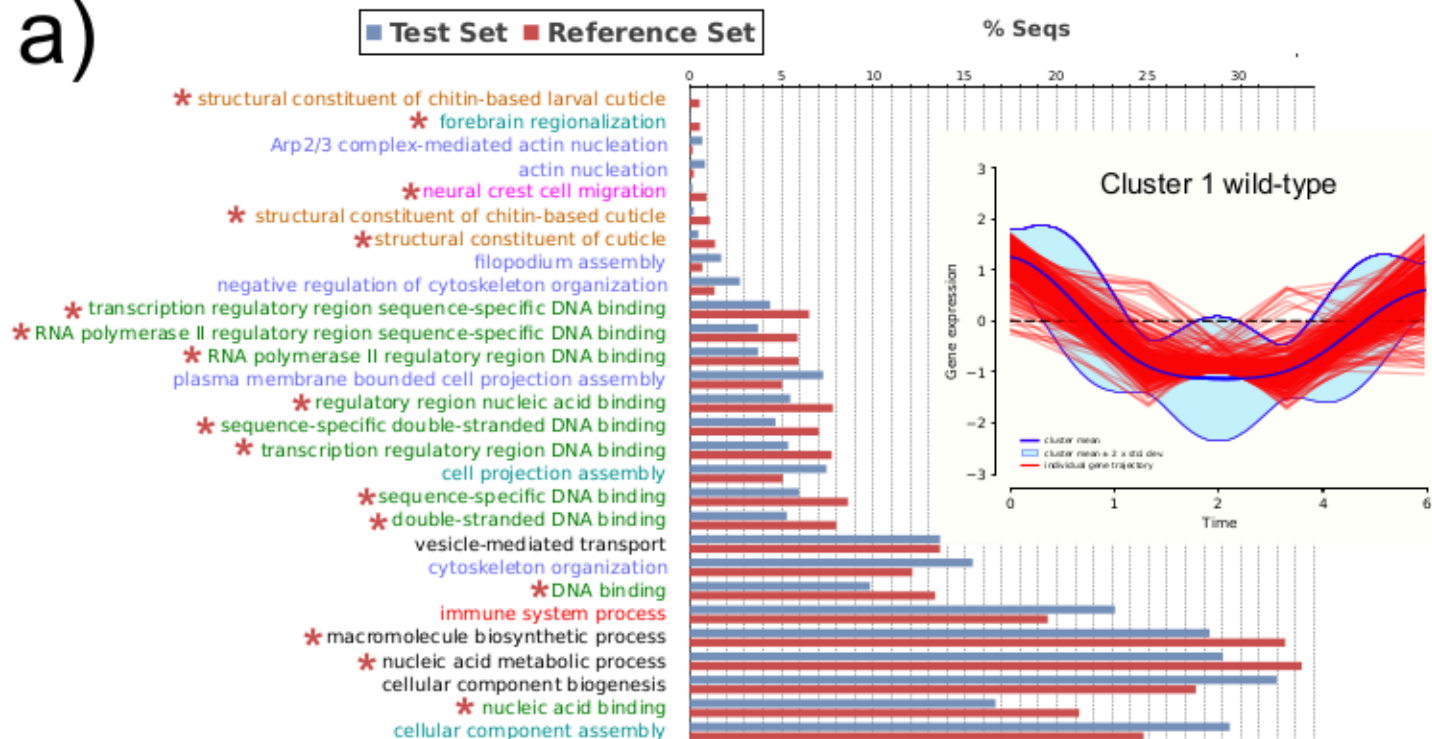

b)

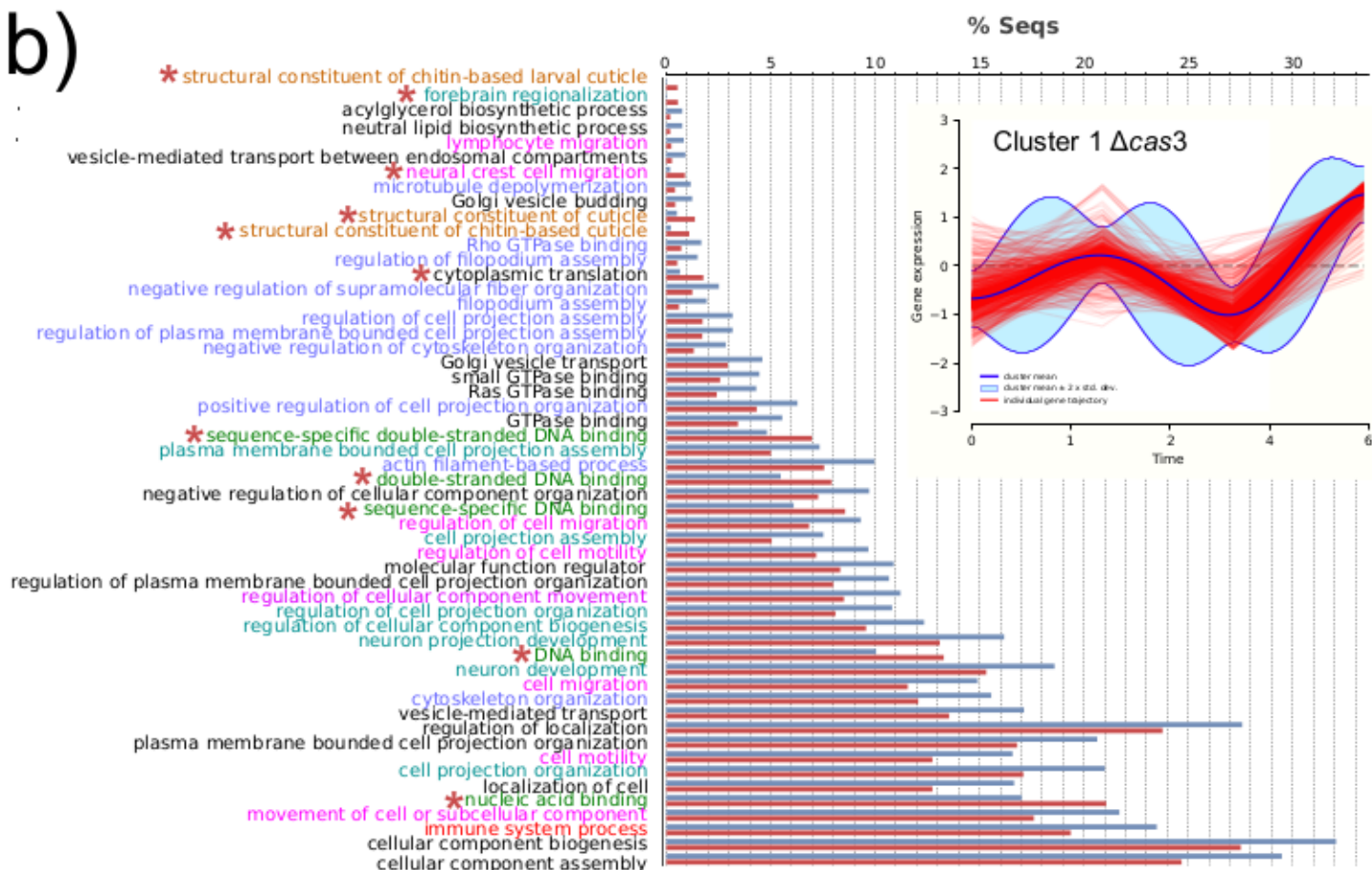

c)

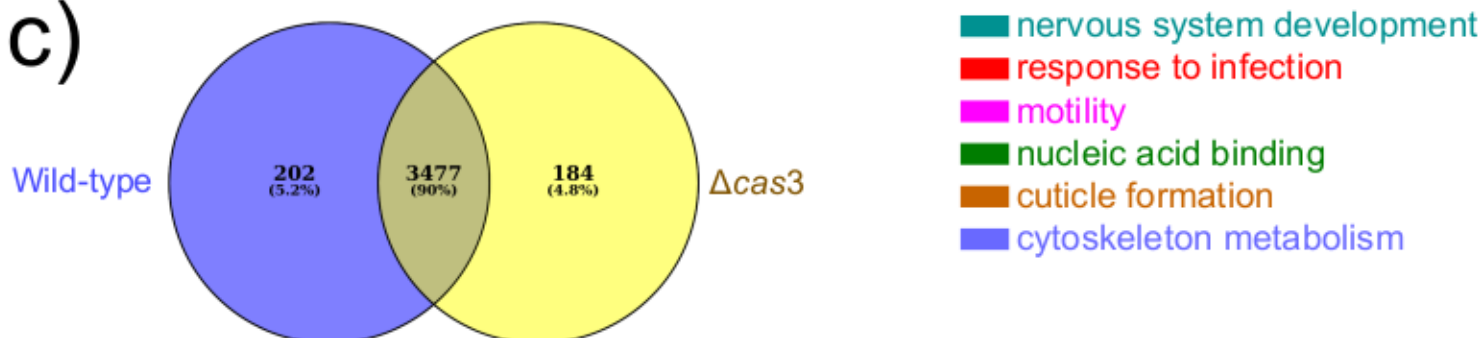

Supplement: FIG S5 [file mSystems.00852-20-sf005.pdf]
