# Supplementary material for: Virulence of the Pathogen Porphyromonas gingivalis Is Controlled by the CRISPR-Cas Protein Cas3
Source: mSystems. 2020 Sep 29;5(5):e00852-20. doi: 10.1128/mSystems.00852-20 (PMC7527141; doi:10.1128/mSystems.00852-20)
Supplement: FIG S4 [file mSystems.00852-20-sf004.pdf]

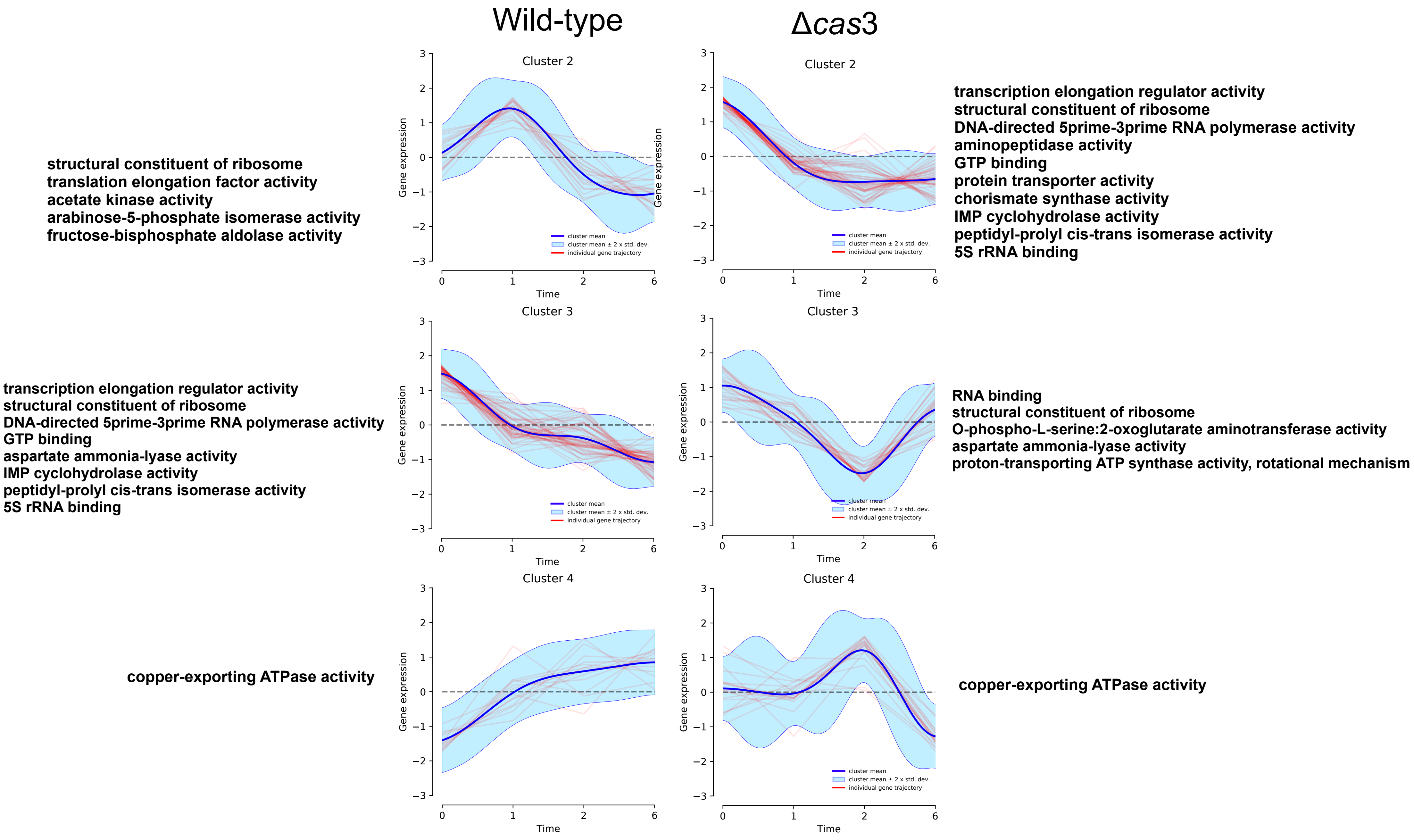
Wild-type
Δcas3
Cluster 2
Cluster 3
Cluster 4
Gene expression
Time
cluster mean
cluster mean ± 2 x std. dev.
individual gene trajectory
structural constituent of ribosome
translation elongation factor activity
acetate kinase activity
arabinose-5-phosphate isomerase activity
fructose-bisphosphate aldolase activity
transcription elongation regulator activity
structural constituent of ribosome
DNA-directed 5prime-3prime RNA polymerase activity
aminopeptidase activity
GTP binding
protein transporter activity
chorismate synthase activity
IMP cyclohydrolase activity
peptidyl-prolyl cis-trans isomerase activity
5S rRNA binding
transcription elongation regulator activity
structural constituent of ribosome
DNA-directed 5prime-3prime RNA polymerase activity
GTP binding
aspartate ammonia-lyase activity
IMP cyclohydrolase activity
peptidyl-prolyl cis-trans isomerase activity
5S rRNA binding
RNA binding
structural constituent of ribosome
O-phospho-L-serine:2-oxoglutarate aminotransferase activity
aspartate ammonia-lyase activity
proton-transporting ATP synthase activity, rotational mechanism
copper-exporting ATPase activity
copper-exporting ATPase activity
