# Supplementary material for: Virulence of the Pathogen Porphyromonas gingivalis Is Controlled by the CRISPR-Cas Protein Cas3
Source: mSystems. 2020 Sep 29;5(5):e00852-20. doi: 10.1128/mSystems.00852-20 (PMC7527141; doi:10.1128/mSystems.00852-20)
Supplement: FIG S6 [file mSystems.00852-20-sf006.pdf]

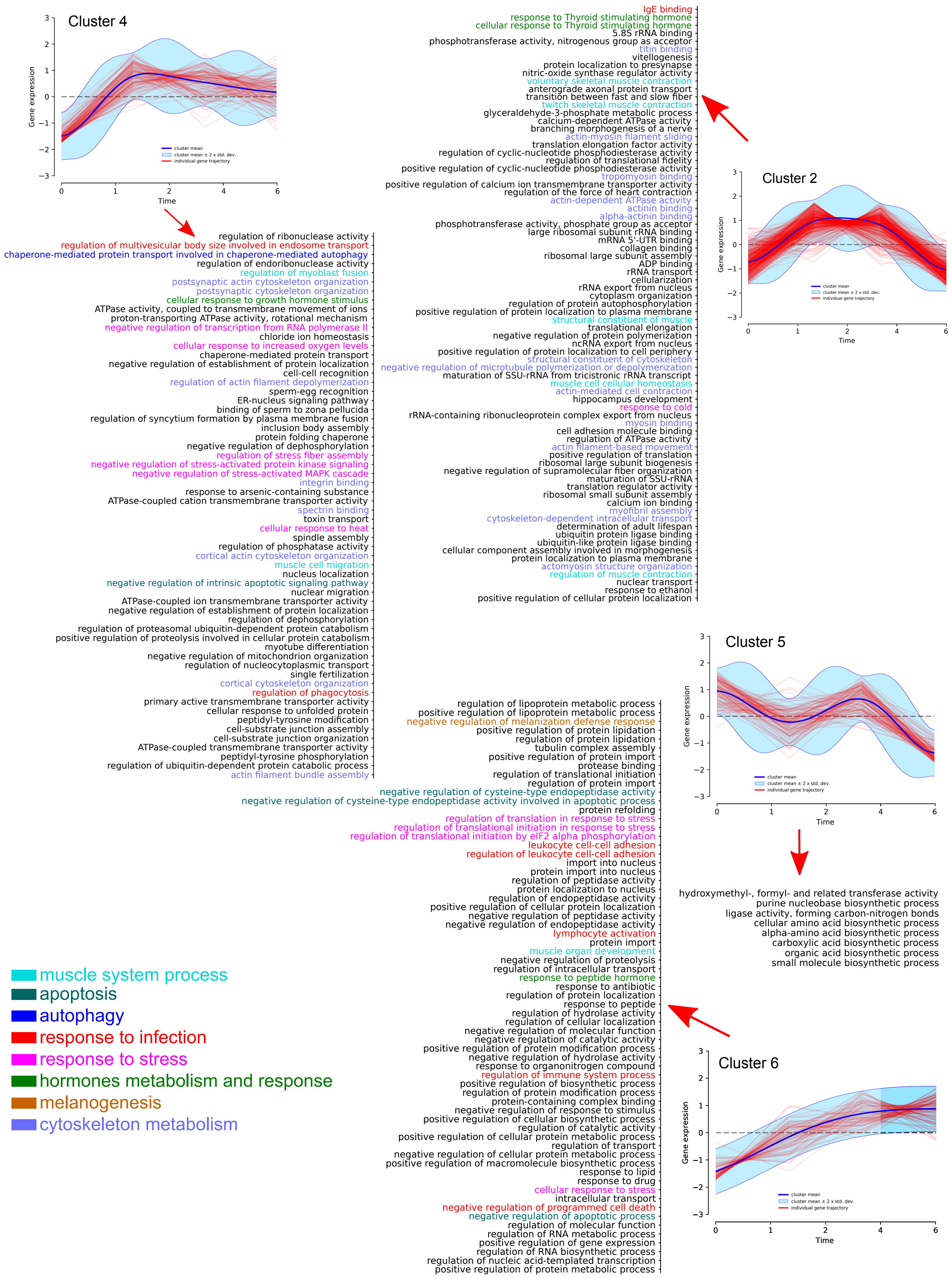

Cluster 4
Gene expression
Time
cluster mean
cluster mean ± 2 x std. dev.
individual gene trajectory
regulation of ribonuclease activity
regulation of multivesicular body size involved in endosome transport
chaperone-mediated protein transport involved in chaperone-mediated autophagy
regulation of endoribonuclease activity
regulation of myoblast fusion
postsynaptic actin cytoskeleton organization
postsynaptic cytoskeleton organization
cellular response to growth hormone stimulus
ATPase activity, coupled to transmembrane movement of ions
proton-transporting ATPase activity, rotational mechanism
negative regulation of transcription from RNA polymerase II
chloride ion homeostasis
cellular response to increased oxygen levels
chaperone-mediated protein transport
negative regulation of establishment of protein localization
cell-cell recognition
regulation of actin filament depolymerization
sperm-egg recognition
ER-nucleus signaling pathway
binding of sperm to zona pellucida
regulation of syncytium formation by plasma membrane fusion
inclusion body assembly
protein folding chaperone
negative regulation of dephosphorylation
regulation of stress fiber assembly
negative regulation of stress-activated protein kinase signaling
negative regulation of stress-activated MAPK cascade
integrin binding
response to arsenic-containing substance
ATPase-coupled cation transmembrane transporter activity
spectrin binding
toxin transport
cellular response to heat
spindle assembly
regulation of phosphatase activity
cortical actin cytoskeleton organization
muscle cell migration
nucleus localization
negative regulation of intrinsic apoptotic signaling pathway
nuclear migration
ATPase-coupled ion transmembrane transporter activity
negative regulation of establishment of protein localization
regulation of dephosphorylation
regulation of proteasomal ubiquitin-dependent protein catabolism
positive regulation of proteolysis involved in cellular protein catabolism
myotube differentiation
negative regulation of mitochondrion organization
regulation of nucleocytoplasmic transport
single fertilization
cortical cytoskeleton organization
regulation of phagocytosis
primary active transmembrane transporter activity
cellular response to unfolded protein
peptidyl-tyrosine modification
cell-substrate junction assembly
cell-substrate junction organization
ATPase-coupled transmembrane transporter activity
peptidyl-tyrosine phosphorylation
regulation of ubiquitin-dependent protein catabolic process
actin filament bundle assembly
IgE binding
response to Thyroid stimulating hormone
cellular response to Thyroid stimulating hormone
5.8S rRNA binding
phosphotransferase activity, nitrogenous group as acceptor
titin binding
vitellogenesis
protein localization to presynapse
nitric-oxide synthase regulator activity
voluntary skeletal muscle contraction
anterograde axonal protein transport
transition between fast and slow fiber
twitch skeletal muscle contraction
glyceraldehyde-3-phosphate metabolic process
calcium-dependent ATPase activity
branching morphogenesis of a nerve
actin-myosin filament sliding
translation elongation factor activity
regulation of cyclic-nucleotide phosphodiesterase activity
regulation of translational fidelity
positive regulation of cyclic-nucleotide phosphodiesterase activity
tropomyosin binding
positive regulation of calcium ion transmembrane transporter activity
regulation of the force of heart contraction
actin-dependent ATPase activity
actinin binding
alpha-actinin binding
phosphotransferase activity, phosphate group as acceptor
large ribosomal subunit rRNA binding
mRNA 5'-UTR binding
collagen binding
ribosomal large subunit assembly
ADP binding
rRNA transport
cellularization
rRNA export from nucleus
cytoplasm organization
regulation of protein autophosphorylation
positive regulation of protein localization to plasma membrane
structural constituent of muscle
translational elongation
negative regulation of protein polymerization
ncRNA export from nucleus
positive regulation of protein localization to cell periphery
structural constituent of cytoskeleton
negative regulation of microtubule polymerization or depolymerization
maturation of SSU-rRNA from tricistronic rRNA transcript
muscle cell cellular homeostasis
actin-mediated cell contraction
hippocampus development
response to cold
rRNA-containing ribonucleoprotein complex export from nucleus
myosin binding
cell adhesion molecule binding
regulation of ATPase activity
actin filament-based movement
positive regulation of translation
ribosomal large subunit biogenesis
negative regulation of supramolecular fiber organization
maturation of SSU-rRNA
translation regulator activity
ribosomal small subunit assembly
calcium ion binding
myofibril assembly
cytoskeleton-dependent intracellular transport
determination of adult lifespan
ubiquitin protein ligase binding
ubiquitin-like protein ligase binding
cellular component assembly involved in morphogenesis
protein localization to plasma membrane
actomyosin structure organization
regulation of muscle contraction
nuclear transport
response to ethanol
positive regulation of cellular protein localization
Cluster 2
Gene expression
Time
cluster mean
cluster mean ± 2 x std. dev.
individual gene trajectory
Cluster 5
Gene expression
Time
cluster mean
cluster mean ± 2 x std. dev.
individual gene trajectory
regulation of lipoprotein metabolic process
positive regulation of lipoprotein metabolic process
negative regulation of melanization defense response
positive regulation of protein lipidation
regulation of protein lipidation
tubulin complex assembly
positive regulation of protein import
protease binding
regulation of translational initiation
regulation of protein import
negative regulation of cysteine-type endopeptidase activity
negative regulation of cysteine-type endopeptidase activity involved in apoptotic process
protein refolding
regulation of translation in response to stress
regulation of translational initiation in response to stress
regulation of translational initiation by eIF2 alpha phosphorylation
leukocyte cell-cell adhesion
regulation of leukocyte cell-cell adhesion
import into nucleus
protein import into nucleus
regulation of peptidase activity
protein localization to nucleus
regulation of endopeptidase activity
positive regulation of cellular protein localization
negative regulation of peptidase activity
negative regulation of endopeptidase activity
lymphocyte activation
protein import
muscle organ development
negative regulation of proteolysis
regulation of intracellular transport
response to peptide hormone
response to antibiotic
regulation of protein localization
response to peptide
regulation of hydrolase activity
regulation of cellular localization
negative regulation of molecular function
negative regulation of catalytic activity
positive regulation of protein modification process
negative regulation of hydrolase activity
response to organonitrogen compound
regulation of immune system process
positive regulation of biosynthetic process
regulation of protein modification process
protein-containing complex binding
negative regulation of response to stimulus
positive regulation of cellular biosynthetic process
regulation of catalytic activity
positive regulation of cellular protein metabolic process
regulation of transport
negative regulation of cellular protein metabolic process
positive regulation of macromolecule biosynthetic process
response to lipid
response to drug
cellular response to stress
intracellular transport
negative regulation of programmed cell death
negative regulation of apoptotic process
regulation of molecular function
regulation of RNA metabolic process
positive regulation of gene expression
regulation of RNA biosynthetic process
regulation of nucleic acid-templated transcription
positive regulation of protein metabolic process
hydroxymethyl-, formyl- and related transferase activity
purine nucleobase biosynthetic process
ligase activity, forming carbon-nitrogen bonds
cellular amino acid biosynthetic process
alpha-amino acid biosynthetic process
carboxylic acid biosynthetic process
organic acid biosynthetic process
small molecule biosynthetic process
Cluster 6
Gene expression
Time
cluster mean
cluster mean ± 2 x std. dev.
individual gene trajectory
muscle system process
apoptosis
autophagy
response to infection
response to stress
hormones metabolism and response
melanogenesis
cytoskeleton metabolism
