# Supplementary material for: Virulence of the Pathogen Porphyromonas gingivalis Is Controlled by the CRISPR-Cas Protein Cas3
Source: mSystems. 2020 Sep 29;5(5):e00852-20. doi: 10.1128/mSystems.00852-20 (PMC7527141; doi:10.1128/mSystems.00852-20)
Supplement: FIG S7 [file mSystems.00852-20-sf007.pdf]

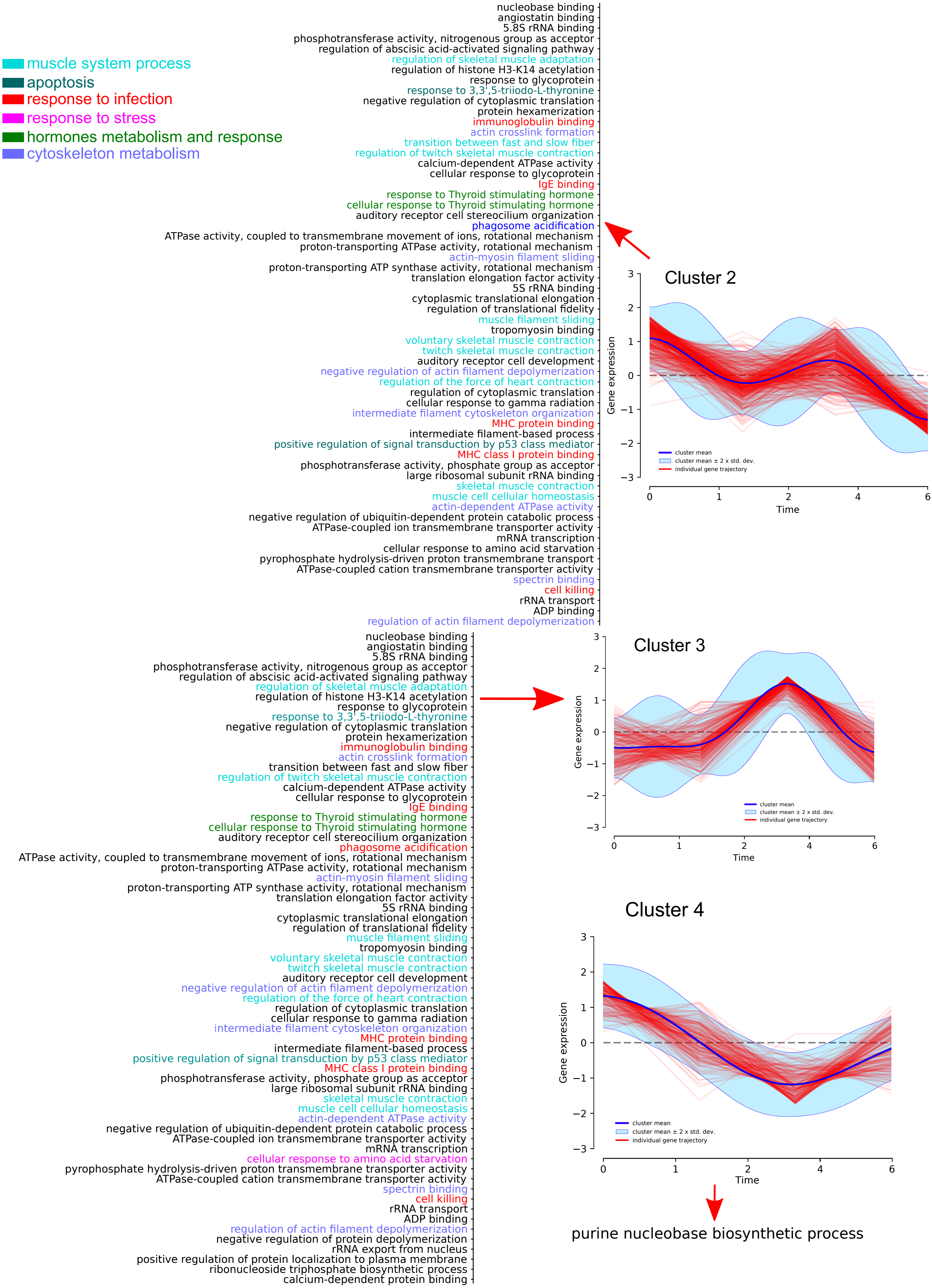

muscle system process
apoptosis
response to infection
response to stress
hormones metabolism and response
cytoskeleton metabolism
nucleobase binding
angiostatin binding
5.8S rRNA binding
phosphotransferase activity, nitrogenous group as acceptor
regulation of abscisic acid-activated signaling pathway
regulation of skeletal muscle adaptation
regulation of histone H3-K14 acetylation
response to glycoprotein
response to 3,3',5-triiodo-L-thyronine
negative regulation of cytoplasmic translation
protein hexamerization
immunoglobulin binding
actin crosslink formation
transition between fast and slow fiber
regulation of twitch skeletal muscle contraction
calcium-dependent ATPase activity
cellular response to glycoprotein
IgE binding
response to Thyroid stimulating hormone
cellular response to Thyroid stimulating hormone
auditory receptor cell stereocilium organization
phagosome acidification
ATPase activity, coupled to transmembrane movement of ions, rotational mechanism
proton-transporting ATPase activity, rotational mechanism
actin-myosin filament sliding
proton-transporting ATP synthase activity, rotational mechanism
translation elongation factor activity
5S rRNA binding
cytoplasmic translational elongation
regulation of translational fidelity
muscle filament sliding
tropomyosin binding
voluntary skeletal muscle contraction
twitch skeletal muscle contraction
auditory receptor cell development
negative regulation of actin filament depolymerization
regulation of the force of heart contraction
regulation of cytoplasmic translation
cellular response to gamma radiation
intermediate filament cytoskeleton organization
MHC protein binding
intermediate filament-based process
positive regulation of signal transduction by p53 class mediator
MHC class I protein binding
phosphotransferase activity, phosphate group as acceptor
large ribosomal subunit rRNA binding
skeletal muscle contraction
muscle cell cellular homeostasis
actin-dependent ATPase activity
negative regulation of ubiquitin-dependent protein catabolic process
ATPase-coupled ion transmembrane transporter activity
mRNA transcription
cellular response to amino acid starvation
pyrophosphate hydrolysis-driven proton transmembrane transport
ATPase-coupled cation transmembrane transporter activity
spectrin binding
cell killing
rRNA transport
ADP binding
regulation of actin filament depolymerization
Cluster 2
Gene expression
Time
cluster mean
cluster mean ± 2 x std. dev.
individual gene trajectory
nucleobase binding
angiostatin binding
5.8S rRNA binding
phosphotransferase activity, nitrogenous group as acceptor
regulation of abscisic acid-activated signaling pathway
regulation of skeletal muscle adaptation
regulation of histone H3-K14 acetylation
response to glycoprotein
response to 3,3',5-triiodo-L-thyronine
negative regulation of cytoplasmic translation
protein hexamerization
immunoglobulin binding
actin crosslink formation
transition between fast and slow fiber
regulation of twitch skeletal muscle contraction
calcium-dependent ATPase activity
cellular response to glycoprotein
IgE binding
response to Thyroid stimulating hormone
cellular response to Thyroid stimulating hormone
auditory receptor cell stereocilium organization
phagosome acidification
ATPase activity, coupled to transmembrane movement of ions, rotational mechanism
proton-transporting ATPase activity, rotational mechanism
actin-myosin filament sliding
proton-transporting ATP synthase activity, rotational mechanism
translation elongation factor activity
5S rRNA binding
cytoplasmic translational elongation
regulation of translational fidelity
muscle filament sliding
tropomyosin binding
voluntary skeletal muscle contraction
twitch skeletal muscle contraction
auditory receptor cell development
negative regulation of actin filament depolymerization
regulation of the force of heart contraction
regulation of cytoplasmic translation
cellular response to gamma radiation
intermediate filament cytoskeleton organization
MHC protein binding
intermediate filament-based process
positive regulation of signal transduction by p53 class mediator
MHC class I protein binding
phosphotransferase activity, phosphate group as acceptor
large ribosomal subunit rRNA binding
skeletal muscle contraction
muscle cell cellular homeostasis
actin-dependent ATPase activity
negative regulation of ubiquitin-dependent protein catabolic process
ATPase-coupled ion transmembrane transporter activity
mRNA transcription
cellular response to amino acid starvation
pyrophosphate hydrolysis-driven proton transmembrane transporter activity
ATPase-coupled cation transmembrane transporter activity
spectrin binding
cell killing
rRNA transport
ADP binding
regulation of actin filament depolymerization
negative regulation of protein depolymerization
rRNA export from nucleus
positive regulation of protein localization to plasma membrane
ribonucleoside triphosphate biosynthetic process
calcium-dependent protein binding
Cluster 3
Gene expression
Time
cluster mean
cluster mean ± 2 x std. dev.
individual gene trajectory
Cluster 4
Gene expression
Time
cluster mean
cluster mean ± 2 x std. dev.
individual gene trajectory
purine nucleobase biosynthetic process
