# Supplementary material for: Virulence of the Pathogen Porphyromonas gingivalis Is Controlled by the CRISPR-Cas Protein Cas3
Source: mSystems. 2020 Sep 29;5(5):e00852-20. doi: 10.1128/mSystems.00852-20 (PMC7527141; doi:10.1128/mSystems.00852-20)
Supplement: FIG S8 [file mSystems.00852-20-sf008.pdf]

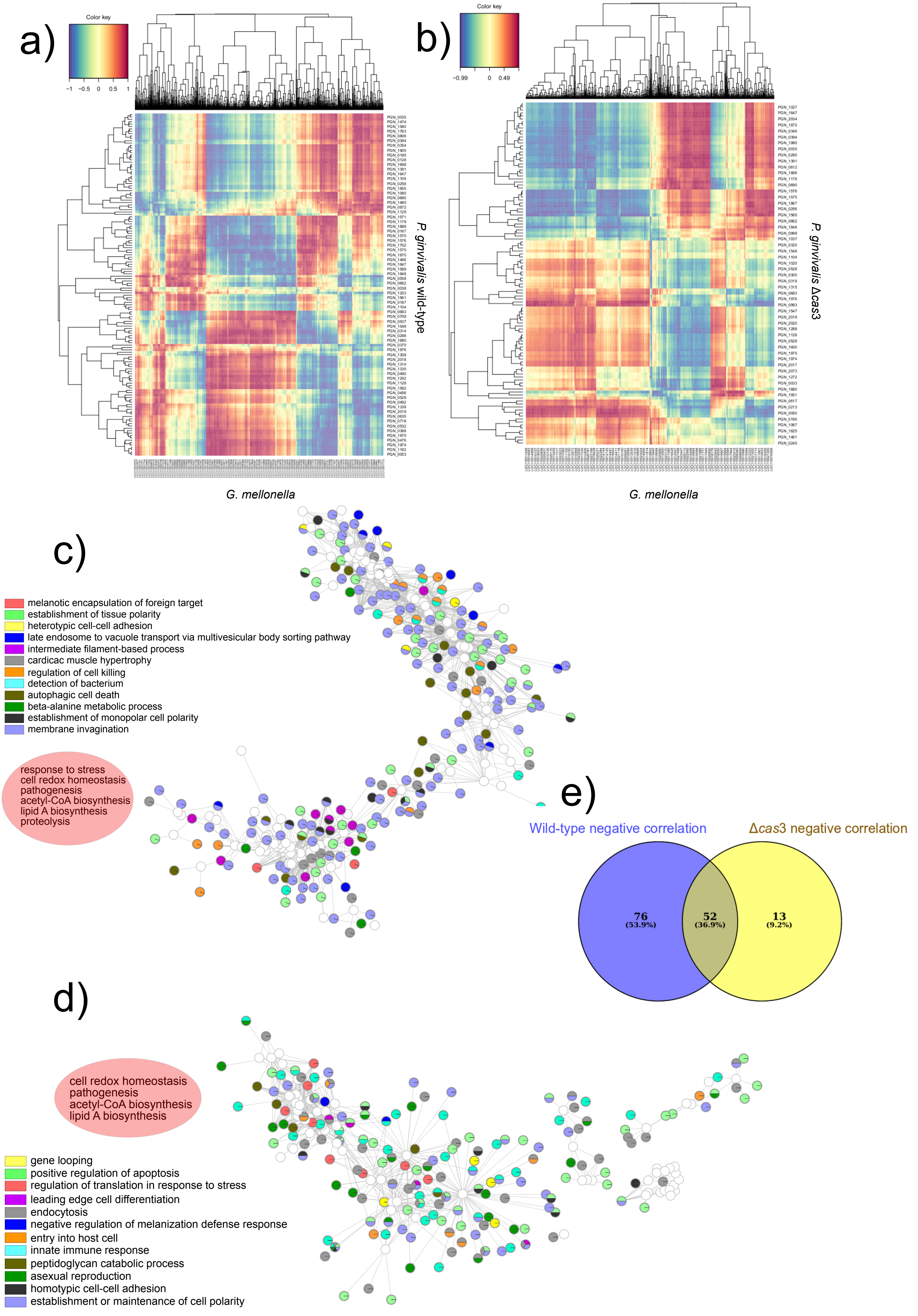

a)
Color key
−1 −0.5 0 0.5 1
P. ginvivalis wild-type
G. mellonella
b)
Color key
−0.99 0 0.49
P. ginvivalis Δcas3
G. mellonella
c)
melanotic encapsulation of foreign target
establishment of tissue polarity
heterotypic cell-cell adhesion
late endosome to vacuole transport via multivesicular body sorting pathway
intermediate filament-based process
cardicac muscle hypertrophy
regulation of cell killing
detection of bacterium
autophagic cell death
beta-alanine metabolic process
establishment of monopolar cell polarity
membrane invagination
response to stress
cell redox homeostasis
pathogenesis
acetyl-CoA biosynthesis
lipid A biosynthesis
proteolysis
e)
Wild-type negative correlation
Δcas3 negative correlation
76
(53.9%)
52
(36.9%)
13
(9.2%)
d)
cell redox homeostasis
pathogenesis
acetyl-CoA biosynthesis
lipid A biosynthesis
gene looping
positive regulation of apoptosis
regulation of translation in response to stress
leading edge cell differentiation
endocytosis
negative regulation of melanization defense response
entry into host cell
innate immune response
peptidoglycan catabolic process
asexual reproduction
homotypic cell-cell adhesion
establishment or maintenance of cell polarity
